# Chimeric allergen receptor regulatory T cells suppress birch pollen allergic airway inflammation

**DOI:** 10.1084/jem.20252201

**Published:** 2026-07-06

**Authors:** Ana Alcaraz-Serna, Noémie Chillier, Aurélien Trompette, Laura Ermellino, Raphaël Porret, Erica Lana, Rebecca Cecchin, Apiha Shanmuganathan, Seher Guney, Oscar Alfageme-Abello, Eleonora Pace, Antonio Mancarella, Jeremy Campos, Mathilde Foglierini, Christophe von Garnier, Craig Fenwick, Laurent Perez, Niki Ubags, Yannick D. Muller

**Affiliations:** 1 Division of Immunology and Allergy, Lausanne University Hospital and University of Lausanne, Lausanne, Switzerland; 2 Division of Pulmonary Medicine, Department of Medicine, Lausanne University Hospital (CHUV), University of Lausanne, Lausanne, Switzerland; 3 Center of Human Immunology Lausanne, Lausanne, Switzerland

## Abstract

Asthma is a deadly chronic respiratory disease affecting over 300 million people. While allergen immunotherapy remains the only disease-modifying treatment, it is poorly applicable for patients with severe asthma. Here, we explored the therapeutic potential of regulatory T cells (Tregs) armed with chimeric allergen receptors—named CAlleR—redirected against the major allergen of birch pollen Bet v1. Four novel anti-Bet v1 antibodies were identified and used to engineer and functionally validate CAlleR. CAlleR Tregs showed specific *in vitro *activation and suppression and significantly reduced the airway hyperresponsiveness in birch pollen–sensitized mice. Mechanistically, CAlleR Tregs migrated to the lungs and mediastinal lymph nodes, interacted with CD11c^+^ dendritic cells, and were activated in a FcγR-dependent manner by cross-presenting Bet v1 stabilized with noncompetitive anti-Bet v1 antibodies. These findings unveil a novel mechanism for targeting soluble antigens and highlight the potential of CAlleR Tregs to prevent and treat severe allergies.

## Teaser

Alcaraz-Serna et al. have engineered regulatory T cells to specifically target the major birch pollen allergen Bet v1. These cells suppressed allergic immune responses and reduced airway inflammation in mice, opening novel strategic avenues for treating allergic asthma.

## Graphical Abstract

**Figure:**
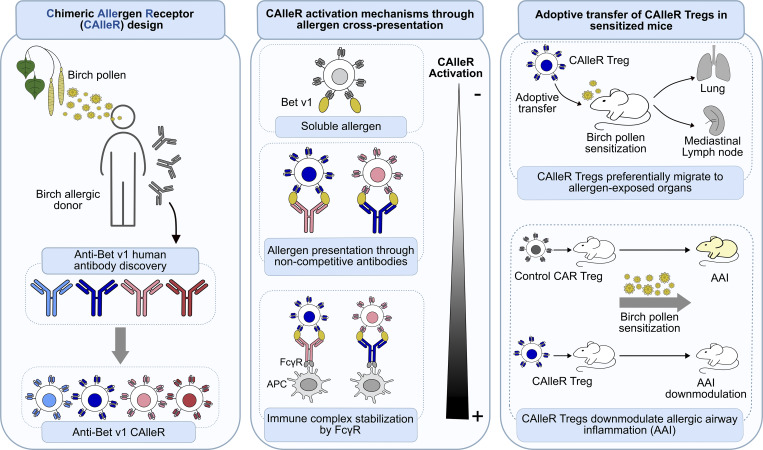

## Introduction

Asthma affects over 300 million people worldwide and remains a deadly disease if insufficiently treated, representing a high burden on individuals, caregivers, and healthcare systems (Papi et al., 2018, Global Initiative for Asthma, 2026). Allergic asthma is driven by an exacerbated type 2 immune response, characterized by the overproduction of IL-4, IL-5, and IL-13 by Th2 cells. These cytokines promote IgE class switching, eosinophil recruitment, and mast cell activation resulting in airway hyperresponsiveness and airway mucus plugging, the principal cause of death in asthma (Maddox and Schwartz, 2002). Its global economic burden is estimated at $80–100 billion per year, with severe cases, which represent 5–10% of asthma patients (Wang et al., 2020) requiring biologic therapies reaching $20,000–40,000 per patient annually (Grønseth and Jansson, 2014; Wang et al., 2023). Despite the approval of several anti-cytokine biologics, allergen immunotherapy (AIT) remains the only disease-modifying therapy (Brusselle and Koppelman, 2022; Cox et al., 2011; Israel and Reddel, 2017).

A major cause of allergic rhinitis and asthma is related to birch sensitization affecting 8–16% of the European population (Biedermann et al., 2019). Birch pollen cross-reacts with a large family of trees including alder, hazel, oak, hornbeam, chestnut, and beech. This birch homologous group shares highly cross-reactive allergens derived from the pathogenesis-related 10 protein (PR-10) family, among which the birch allergen Bet v1 is the immunodominant and most abundant allergenic protein (Schenk et al., 2011). While AIT for birch pollen–associated rhinitis and asthma has been shown to be effective (Wahn et al., 2019), it remains contraindicated in patients with severe and uncontrolled asthma and is poorly effective in polysensitized patients (Cox et al., 2011). This highlights the unmet need for new, safe, and durable treatments for restoring allergen tolerance in severe allergic asthma.

Regulatory T cells (Tregs) are crucial for suppressing type 2 inflammation through multiple modalities including IL-2 consumption, production of IL-10 and TGF-β, and downregulation of dendritic cell immunogenic activities (Akbari et al., 2003). Importantly, Tregs can be expanded *ex vivo* and reinfused with multiple clinical trials evaluating their potential in autoimmune and inflammatory disorders (Raffin et al., 2020). However, Treg therapy has shown only limited efficacy, which has been mostly attributed to the lack of antigen specificity (Baeten et al., 2022). To overcome this limitation, efforts have focused on redirecting Treg specificity by engineering synthetic receptors such as chimeric antigen receptors (CARs) (Baeten et al., 2022).

Herein, we hypothesized that Tregs can be armed with a chimeric allergen receptor (CAlleR) to target allergens such as Bet v1 to reset tolerance against birch pollen–associated allergic diseases. We identified and characterized four novel anti–birch-specific antibodies and generated single-chain variable fragments (scFvs) fused to a CD28-ζ signaling domain. We demonstrated allergen-specific response of CAlleRs. To decipher the physiological mechanisms by which CAlleR activation occurs, we showed that soluble allergens can induce maximal activity of CAlleRs when stabilized by a noncompetitive allergen-specific antibody in a Fcγ receptor (FcγR)–dependent manner. Finally, CAlleR Tregs preserved lung function in birch pollen–sensitized mice.

## Results

### Characterization of four novel high-affinity anti-Bet v1 monoclonal antibodies

**Figure S1.**
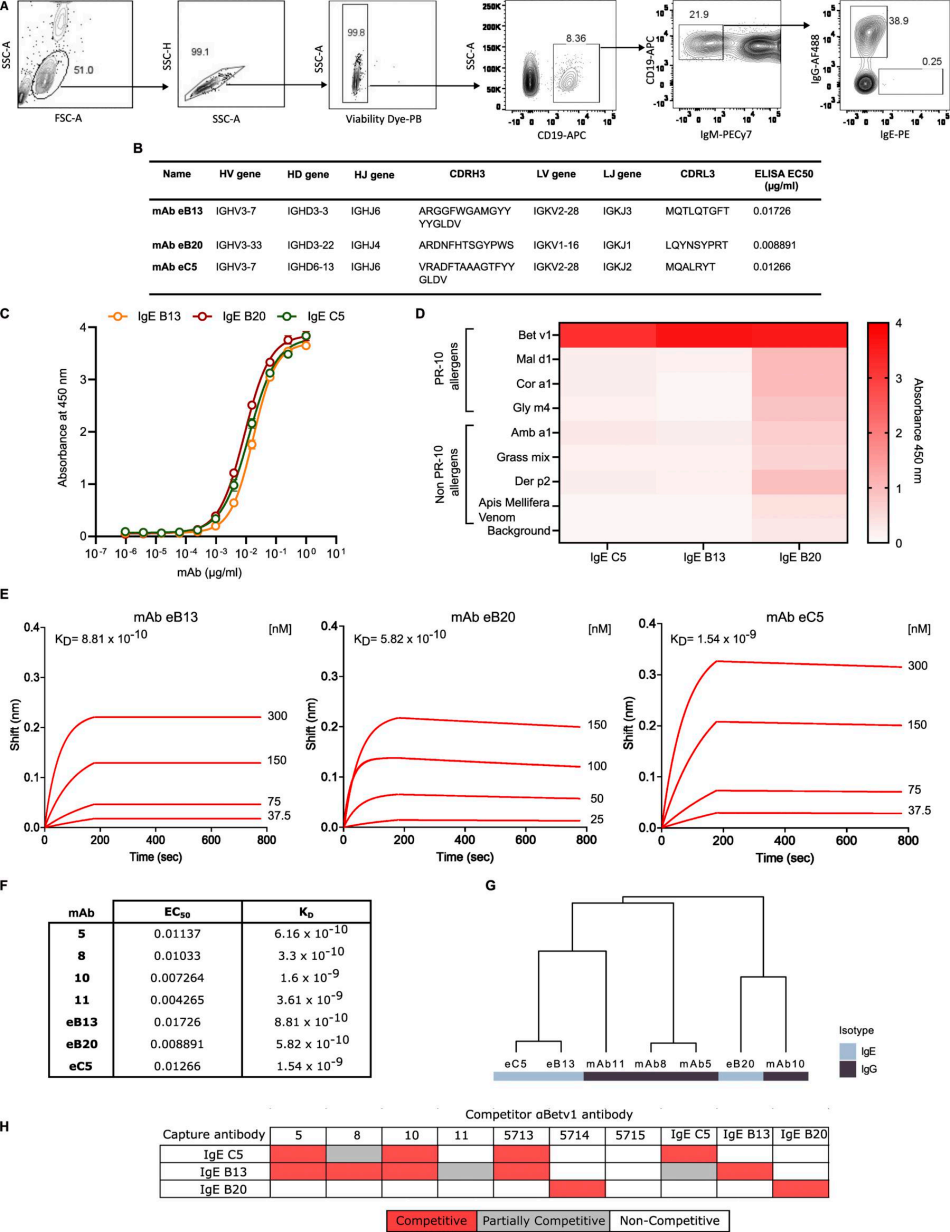
**Human B cell sorting for antibody discovery and characterization of three novel human anti-Bet v1 IgE antibodies. (A)** Gating strategy for the isolation of human CD19^+^IgM^−^IgE^−^IgG^+^ and CD19^+^IgM^−^IgE^+^IgG^− ^sorted cells. **(B)** Characteristics of three human anti-Bet v1 IgE antibodies with variable domain genes (V), (D), and (J) for heavy (H) and light (L) chains, CDR3 amino acid sequences, and EC50 against Bet v1. **(C)** Anti-Bet v1 ELISA of the novel anti-Bet v1 antibodies (*n* = 2). **(D)** Specificity of anti-Bet v1 antibodies to PR-10 and non-PR-10 allergens assessed by ELISA. **(E)** BLI kinetics of each novel antibody immobilized on a protein A–loaded biosensor and Bet v1 in solution. Red lines represent fittings to the sensogram traces. Bet v1 concentrations used for each antibody are reported in nM. **(F)** Comparative table with binding and affinity to Bet v1 of novel IgG and IgE antibodies. **(G)** Hierarchical clustering of antibody variable domain sequences visualized as a dendrogram. Antibody isotypes are indicated alongside the dendrogram (blue: IgE; gray: IgG). **(H)** Competitive binding study between antibodies binding Bet v1 protein–coupled beads. Competitors induced either strong blocking (red boxes), partial competition (gray boxes), or noncompetitive binding (white boxes) with corresponding mAb to Bet v1. Data in C are represented as the mean ± SEM. CDR3, third complementarity-determining region.

**Figure 1.**
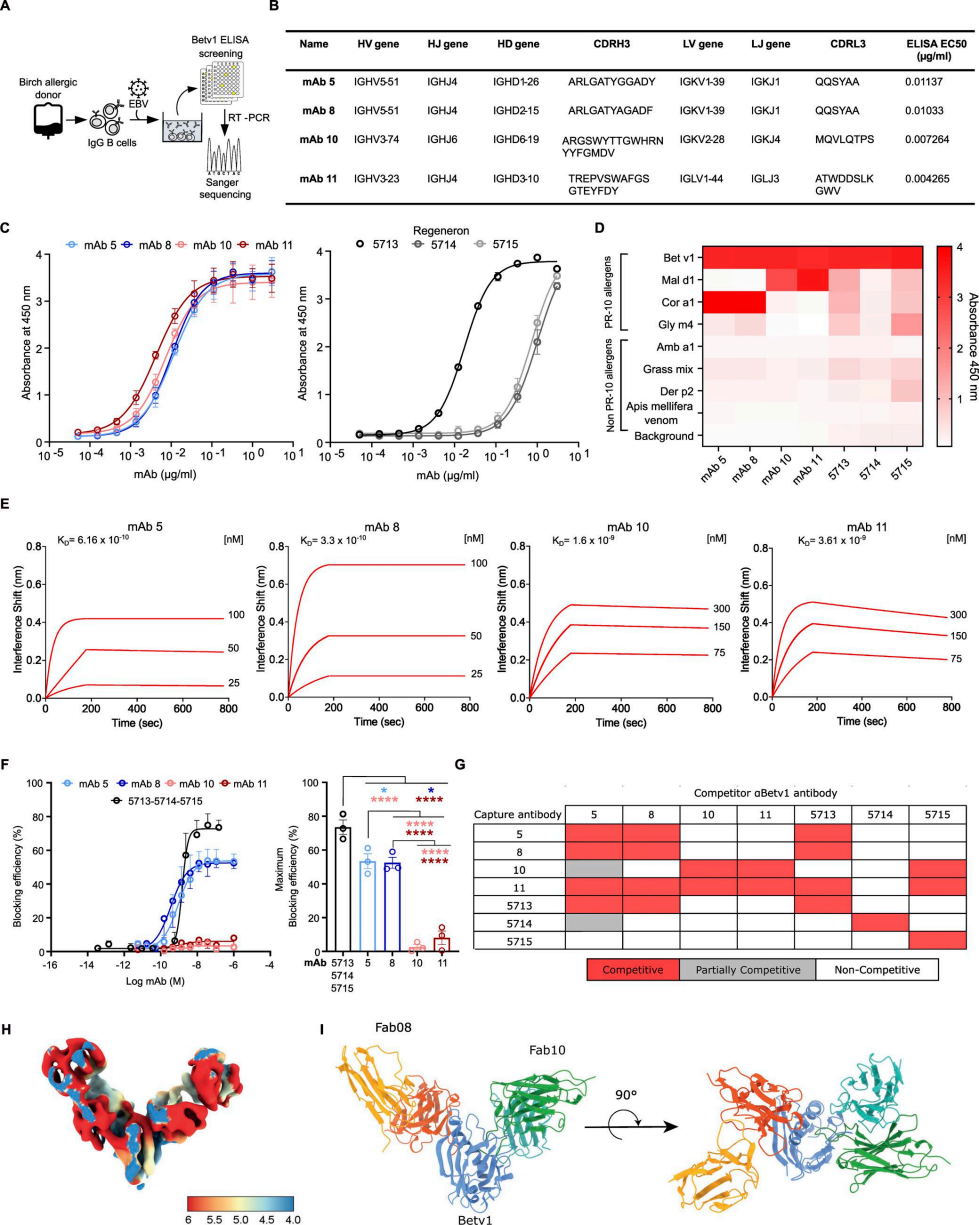
**Discovery and characterization of four novel anti-Bet v1 human antibodies. (A)** Experimental design of human anti-Bet v1 discovery. **(B)** Characteristics of four human anti-Bet v1 antibodies with variable domain genes (V), (D), and (J) for heavy (H) and light (L) chains, CDR3 amino acid sequences, and EC50 against Bet v1. **(C)** Anti-Bet v1 ELISA of the novel anti-Bet v1 antibodies (*n* = 3) and the three anti-Bet v1 Regeneron antibodies (REGN5713, REGN5714, and REGN5715) (*n* = 2). **(D)** Specificity of anti-Bet v1 antibodies to PR-10 and non-PR-10 allergens assessed by ELISA (samples with OD values exceeding the detection range of the plate reader were plotted at the maximal measurable absorbance [OD450 = 4.0]). **(E)** BLI kinetics of each novel antibody immobilized on a protein A–loaded biosensor and Bet v1 in solution. Red lines represent fittings to the sensogram traces. Bet v1 concentrations used for each antibody are reported in nM. **(F)** ELISA testing anti-Bet v1 antibody blocking Bet v1 binding to specific polyclonal IgE (*n* = 3 biologically independent samples). **(G)** Competitive binding study between antibodies binding Bet v1 protein–coupled beads. Competitors induced either strong blocking (red boxes), partial competition (gray boxes), or noncompetitive binding (white boxes) with corresponding mAb to Bet v1. **(H)** Cryo-EM reconstruction of the complex at 4.8 Å resolution. The map is colored according to local resolution. **(I)** Structural model derived from cryo-EM shown from side and top views. Bet v1 is in blue, Fab08 heavy chain in red, Fab08 light chain in orange, Fab10 heavy chain in green, and Fab10 light chain in cyan. Data in C and F are represented as the mean ± SEM. Exact P values were determined by one-way ANOVA with Tukey’s test in F. *P < 0.05; ****P < 0.0001. CDR3, third complementarity-determining region.

CD19^+^IgM^−^IgG^+^ B cells from a birch-allergic donor were sorted and immortalized for single-cell plating (Fig. S1 A). Bet v1 specificity for 5,760 IgG and 20 IgE clones was assessed by ELISA. We could identify four IgG and three IgE Bet v1–specific clones after Sanger sequencing of the heavy variable and light variable gene pairs (Fig. 1, A and B; and Fig. S1 B). Two of the four IgG clones (monoclonal antibody [mAb] 5 and mAb 8) shared the same V gene, IGHV5-51 for the heavy and IGKV1-39 for the light but had different complementarity-determining region 3 (CDRH3) (Fig. 1 B). Similarly, two of the three IgE clones (eB13 and eC5) shared the same V genes for the heavy (IGHV3-7) and the light (IGKV2-28) chains with different CDRH3 (Fig. S1 B). To validate the antibody specificity, we produced all selected mAbs, as well as three previously reported control anti-Bet v1 mAbs (REGN5713, REGN5714, and REGN5715) (Atanasio et al., 2022). Bet v1–specific IgG (5, 8, 10, 11) and IgE (eB13, eB20, and eC5) clones showed similar half maximal effective concentration (Fig. 1, B and C; and Fig. S1, B and C).

To further evaluate the cross-reactivity of our antibodies against the PR-10 family, we tested the binding specificity to Cor a1 (homology to Bet v1 72%), Mal d1 (homology to Bet v1 58%), and Gly m4 (homology to Bet v1 47%). Interestingly, clones 5 and 8 cross-reacted with Cor a1 and clones 10 and 11 cross-reacted with Mal d1, while the remaining only reacted against Bet v1. Importantly, none of the antibodies bound unrelated allergens (Fig. 1 D and Fig. S1 D). We next assessed their binding affinity to Bet v1 by the biolayer interferometry (BLI) assay. Clones 5, 8, eB13, and eB20 showed higher association and almost no dissociation compared with mAb 10, 11, and eC5 (Fig. 1 E and Fig. S1 E). Similarities between mAbs 5 and 8 and between eC5 and eB13 were further supported by hierarchical clustering of antibody sequences (Fig. S1 G). Considering that the cross-recognition of homologous PR-10 allergens represents a potential advantage, we further selected clones 5, 8, 10, and 11 for the downstream analysis.

We next tested the neutralization capacity of the antibodies to block Bet v1–specific IgE binding from three birch-allergic donors. Clones 5 and 8 showed the strongest blocking capacity individually. Despite the high affinity of mAbs 10 and 11, they showed lower blocking effect (Fig. 1 F). Epitope binning by cross-competition binding to Bet v1 revealed that mAbs 5 and 8 recognized the same epitope as REGN5713, which partially overlapped with that bound by mAb 11. In contrast, mAb 10 competed for a partially overlapping epitope also shared with mAb 11 (Fig. 1 G). Among the IgE clones, eC5 inhibited binding of mAbs 5, 8, and 10, and REGN5713. Similarly, eB13 competed with these antibodies and partially with mAb 11. In contrast, eB20 did not compete with the other clones for Bet v1 binding, except with REGN5714 (Fig. S1 H). To confirm that mAbs 8 and 10 recognized distinct regions of Bet v1 and to gain further insight into their epitopes, Bet v1 was complexed with threefold molar excess of Fab08 and Fab10 and analyzed by cryo-electron microscopy (cryo-EM). Reconstruction of the Bet v1-Fab08-Fab10 complex was obtained at a resolution of 4.8 Å, allowing Cα positioning (Fig. 1 H). Bet v1 adopted the canonical fold consisting of a seven-stranded antiparallel β-sheet wrapped around a 25-residue-long C-terminal amphipathic α-helix, as previously described (Gajhede et al., 1996). Fab08 sandwiched the C-terminal α-helix, similarly as REGN5713, with interactions mediated almost exclusively by its heavy chain. In contrast, Fab10 bound a distinct epitope consisting of a loop formed by residues 60–65, which lies within the epitope region recognized by REGN5715 (Fig. 1 I) (Atanasio et al., 2022). Overall, the binding, cross-reactivity, and functional properties of these novel anti-Bet v1 IgG mAbs support their further evaluation as scFvs for the design of CAlleRs.

### Activation and proliferation of CAlleR T cells by artificial antigen-presenting cells (APC) expressing Bet v1

**Figure 2.**
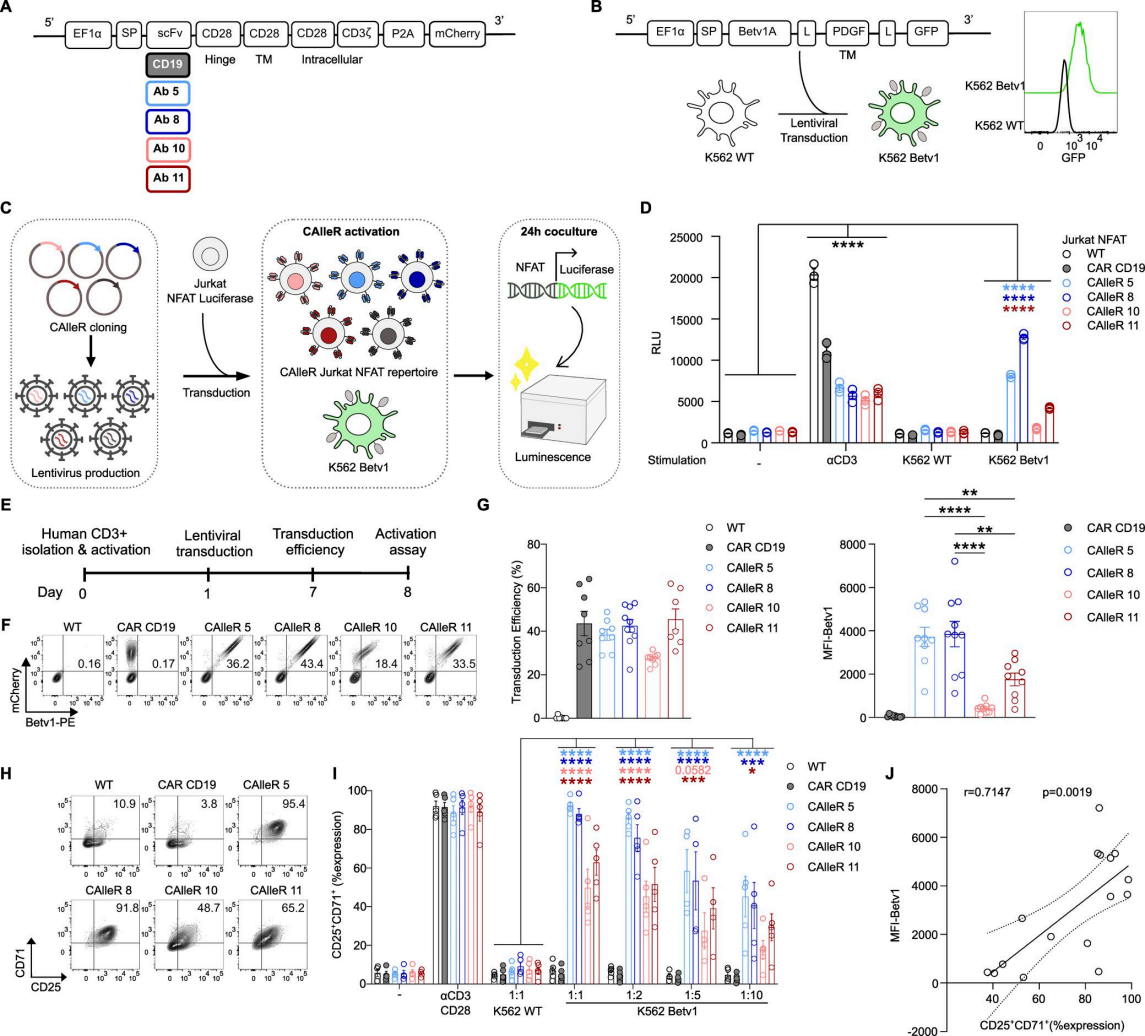
**Validation of four novel anti-Bet v1 CAlleRs. (A)** Anti-Bet v1 CAlleR and anti-CD19 CAR lentiviral constructs. **(B)** Construct of lentivirus encoding surface expression of Bet v1 used to transduce K562 cells and phenotype check by GFP reporter expression. **(C)** Experimental design of a CAlleR screening platform using NFAT-Luciferase CD4^+^ Jurkat cells. **(D)** Luminescence signal measured in RLU of anti-Bet v1 CAlleR and control NFAT cell lines after 24 h coculture with K562 Bet v1 cells (ratio 1:2) (*n* = 3 from three independent experiments). **(E)** Experimental design of human anti-Bet v1 CAlleR T cell production. **(F)** Representative flow cytometry plots showing mCherry reporter expression and binding to Bet v1 of wild-type (WT) and transduced human T cells. **(G)** Cumulative percentages of transduction efficiency (mCherry^+^Bet v1^+^) of CAR CD19 and anti-Bet v1 CAlleRs and MFI of Bet v1 expression (*n* = 7–10 biologically independent experiments). **(H)** Representative flow cytometry plots of CD25 and CD71 expression on T cells after 48-h coculture with K562 WT or K562 Bet v1 (ratio 1:1). **(I)** Cumulative data showing percentages of CD25^+^CD71^+^ T cells after 48-h coculture (*n* = 4–5 biologically independent experiments). **(J)** Correlation between CAlleR Bet v1 binding and activation (CD25^+^CD71^+^ expression) is plotted with best-fit lines using simple linear regression and calculated using Pearson’s correlation coefficient. Data are represented as the mean ± SEM in D, G, and I. Exact P values were determined by two-way ANOVA with Dunnett’s test in D and I, and by one-way ANOVA with Tukey’s test in G. *P < 0.05; **P < 0.01; ***P < 0.001; ****P < 0.0001. RLU, relative light units; MFI, mean fluorescence intensity.

We next generated scFvs derived from our four anti-Bet v1 IgG mAbs and fused them to a CD28 hinge, transmembrane, and costimulatory domains followed by a CD3ζ intracellular signaling domain. Anti-CD19 CAR was used as a control and mCherry as a reporter (Fig. 2 A). To evaluate the functional activity of the CAlleRs, we first engineered artificial K562 cells with a platelet-derived growth factor (PDGF)–truncated receptor covalently linked to the Bet v1 recombinant protein and a GFP molecule on the N and C terminus, respectively (Fig. 2 B). To validate the specificity of the selected CAlleRs, Jurkat NFAT reporter cell lines were transduced with lentiviruses encoding the CAlleRs and cocultured with K562 Bet v1 for 24 h (Fig. 2C). CAlleR 5 and 8 displayed the highest NFAT activity, followed by CAlleR 11 and CAlleR 10 (Fig. 2 D).

Primary human CAlleR T cells were then generated to validate their functionality *in vitro* (Fig. 2 E). Similar transduction efficiencies were obtained (prior to calculation of multiplicity of infection [MOI] = 1) (Fig. 2, F and G). Again, CAlleR 5 and 8 showed higher binding to biotinylated Bet v1 compared with CAlleR 11 and 10 (Fig. 2 G), correlating with CD25 and CD71 upregulation (Fig. 2, H–J).

### Human CAlleR 8 Tregs show the highest *in vitro* suppressive capacity

**Figure 3.**
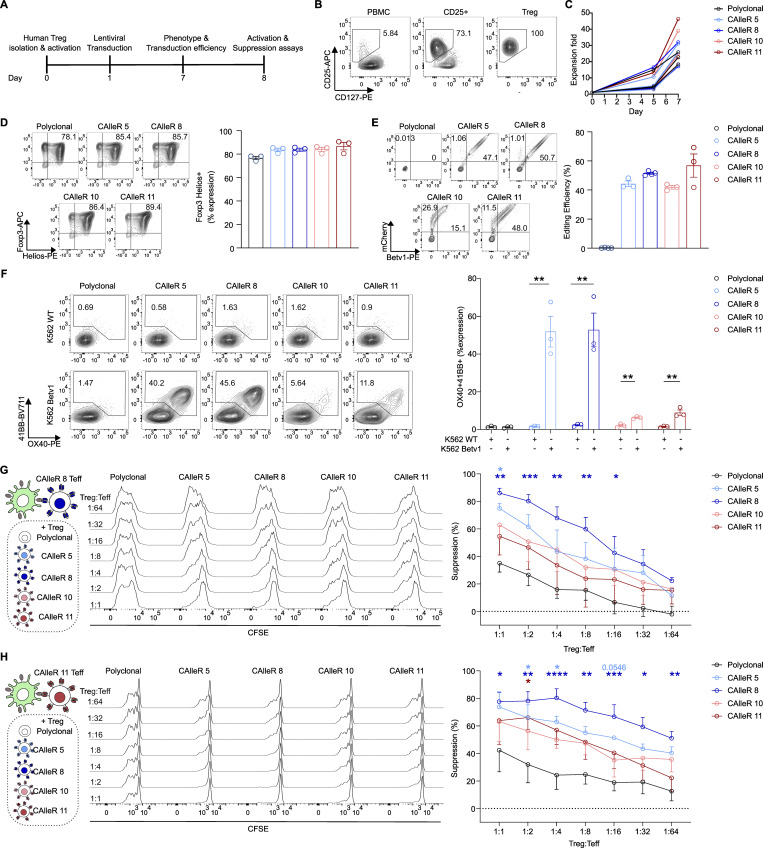
***In vitro* suppression of anti-Bet v1 CAlleR Tregs. (A)** Experimental design of human CAlleR Treg production. **(B)** Representative plots of CD25 and CD127 expression on cells at the different steps of Treg isolation. **(C)** Expansion of Tregs over 7 days of cell culture. **(D)** Representative plots and cumulative data of Foxp3 and Helios expression in Tregs after expansion. **(E)** Representative plots and cumulative data of transduction efficiency of anti-Bet v1 CAlleR Tregs (*n* = 3–4). **(F)** Representative plots and cumulative data of CAlleR Treg activation markers (assessed by 41BB and OX40 expression) after 24-h coculture with K562 WT or K562 Bet v1 at a K562:Treg ratio of 1:5. **(G and H)** Suppressive effect of CAlleR and polyclonal Tregs on (G) CFSE^+^ CAlleR 8 or (H) CFSE^+^ CAlleR 11 Teff. Plots show representative CFSE dilutions of Teff after 96-h coculture with K562 Bet v1 (K562 Bet v1:Teff ratio of 1:10) and different ratios of Tregs and cumulative data showing the percentage of proliferation suppression. Data represent the mean ± SEM from three to four independent experiments. Exact P values were determined by an unpaired *t* test in F and by two-way ANOVA with Dunnett’s test to compare CAlleR with polyclonal Tregs in G and H. *P < 0.05; **P < 0.01; ***P < 0.001; ****P < 0.0001.

The suppressive capacity of CAlleR Tregs was evaluated by applying a similar editing strategy to freshly isolated CD4^+^CD25^+^CD127^low^ Tregs (Fig. 3, A and B). After 7 days of culture, the cells expanded 20–40-fold (Fig. 3 C) and maintained a stable Treg phenotype independently of CAlleR expression (Fig. 3 D). Similar transduction was observed among all CAlleRs (Fig. 3 E). We observed a Bet v1–induced upregulation of early activation markers (OX40 and 41BB) in CAlleR Tregs (Fig. 3 F). Interestingly, the higher activation rates observed with CAlleR 5 and 8 Tregs were consistent with those obtained with T cells (Fig. 2, H and I; and Fig. 3 F). To evaluate their suppressive function, CFSE-labeled CAlleR 8 or CAlleR 11 T cells were cocultured with K562 Bet v1 and different ratios of CAlleR or polyclonally expanded Tregs (Fig. 3, G and H). Overall, CAlleR Tregs exhibited greater suppressive capacity than polyclonal Tregs correlating with the *in vitro* activation levels. CAlleR 8 Tregs exhibited the highest suppressive capacity for both CAlleR 8 and 11 T cells. We therefore selected this candidate for the *in vivo* studies.

### Soluble Bet v1 is cross-presented to CAlleRs by noncompetitive Bet v1–specific antibodies

**Figure 4.**
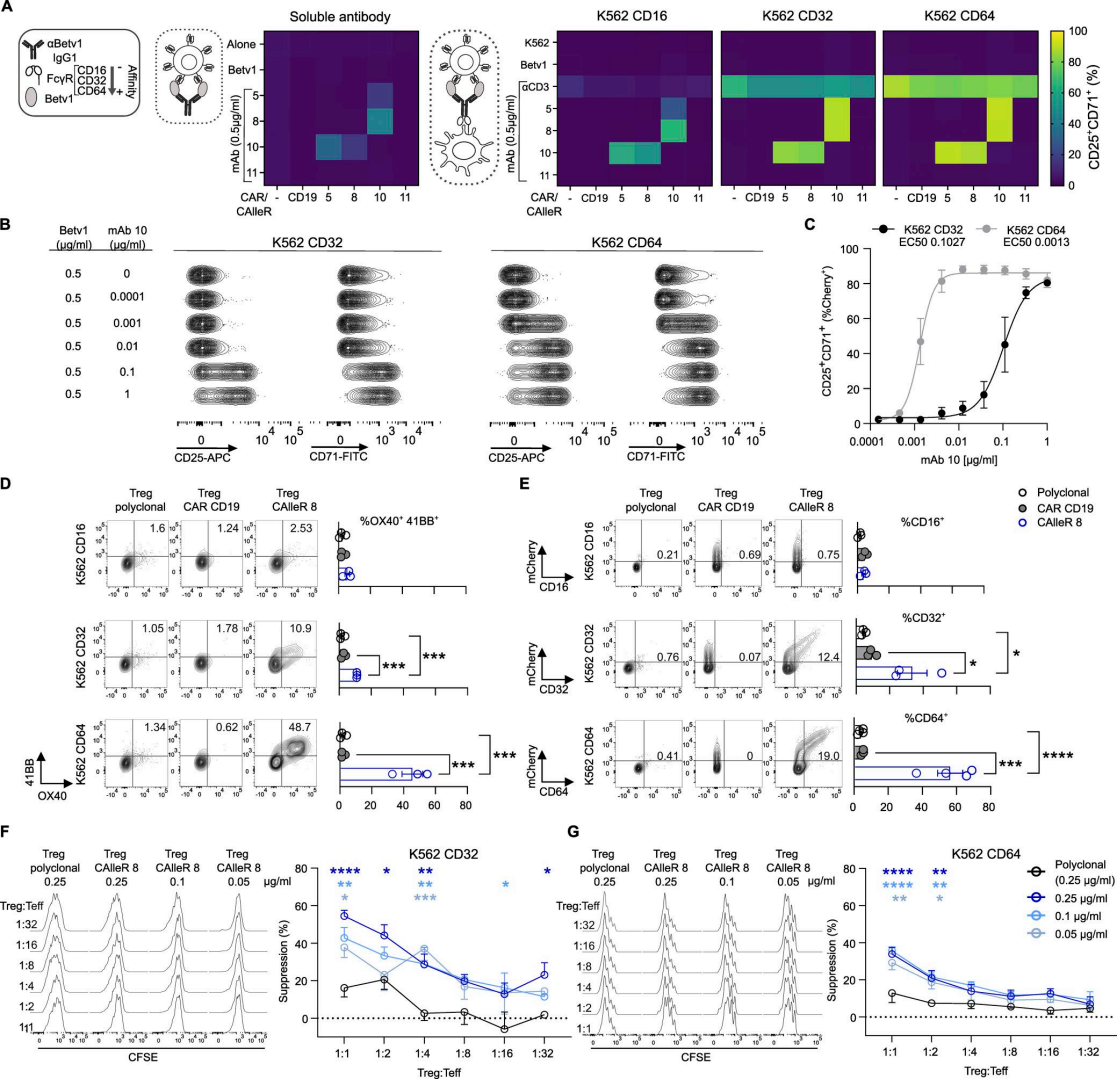
**FcγR-dependent antibody-mediated activation of CAlleRs. (A)** Heatmaps showing mean expression of CD25 and CD71 activation markers on CAlleR T cells upon 48-h coculture with Bet v1 and anti-Bet v1 antibodies with or without K562 expressing three different FcγRs. **(B)** Representative plots of CD25 and CD71 expression on CAlleR 8 T cells upon 48-h coculture (*n* = 3) with different concentrations of mAb 10 and Bet v1 (0.5 μg/ml) in the presence of K562 expressing CD32 or CD64. **(C)** Dose–response curves with EC50 of CAlleR 8 T cell activation in the presence of K562 expressing CD32 or CD64, Bet v1 (0.5 μg/ml), and different concentrations of mAb 10 (*n* = 3). **(D)** Representative flow cytometry plots and cumulative data of 41BB and OX40 expression on CAlleR 8 Treg after 24-h coculture with K562 expressing different FcγRs, mAb 10 (1 μg/ml), and Bet v1 (0.5 μg/ml) (*n* = 3). **(E)** CAlleR 8–mediated trogocytosis of FcγR on Tregs after 24-h coculture with different K562, Bet v1 (0.5 μg/ml), and mAb 10 (1 μg/ml) (*n* = 3). **(F and G)** Suppressive effect of CAlleR 8 and polyclonal Tregs on CAlleR 8 Teff upon 96-h coculture with K562 expressing either (F) CD32 or (G) CD64 (K562:Teff ratio of 1:10), Bet v1 (0.5 μg/ml), and different concentrations of mAb 10. Plots show representative CFSE dilutions of CAlleR 8 Teff and cumulative data of the percentage of proliferation suppression. In A–E, coculture of K562 with T or Tregs was performed at a 1:5 ratio. Data represent the mean ± SEM from three to four independent experiments. P values were determined by one-way ANOVA with Tukey’s multiple comparison test in D and E and by two-way ANOVA with Dunnett’s test to compare CAlleR 8 with polyclonal Tregs in F and G. *P < 0.05; **P < 0.01; ***P < 0.001; ****P < 0.0001.

**Figure S2.**
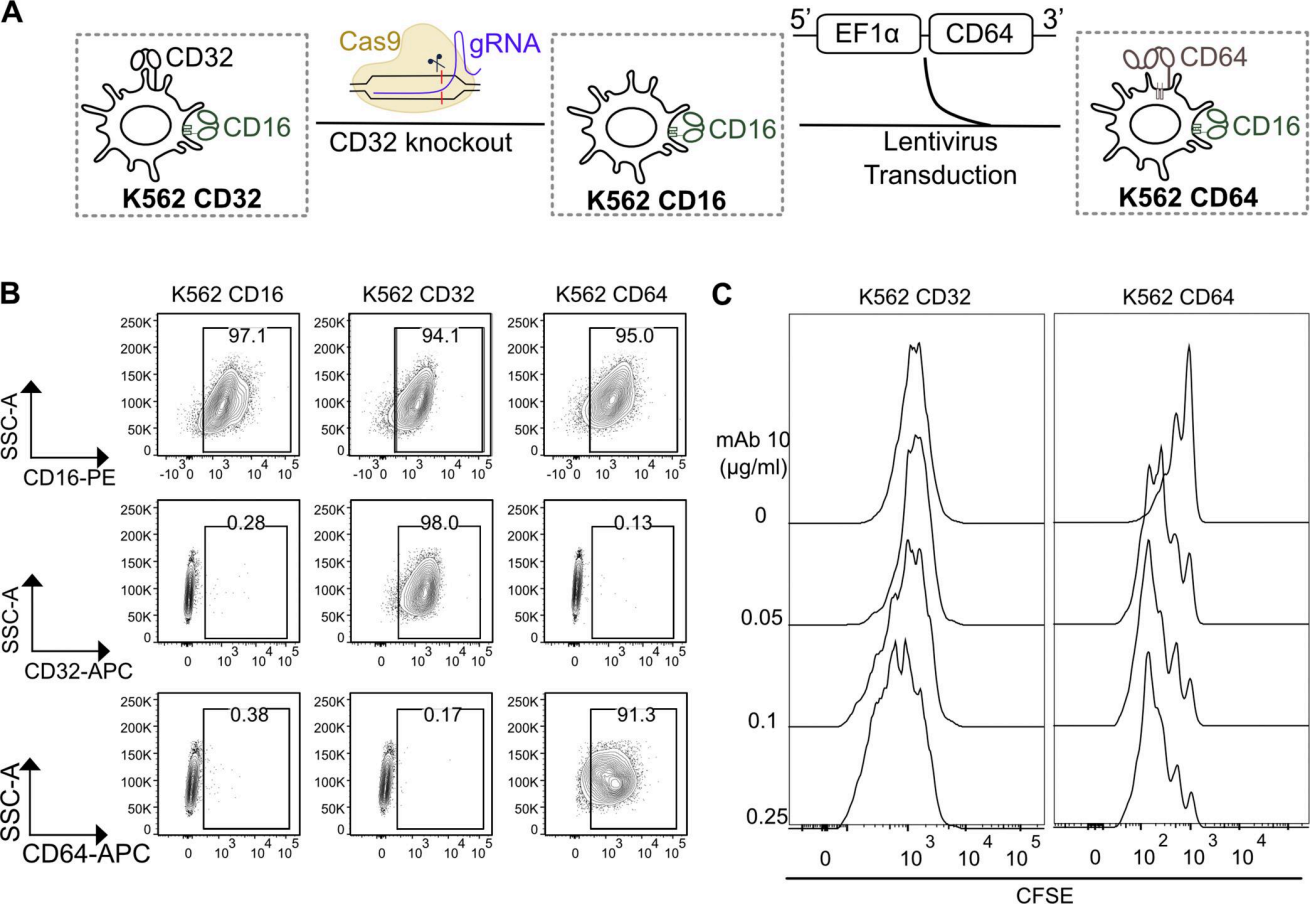
**Generation and validation of K562 cell lines expressing different FcγRs. (A)** Transgenic K562 cell line generation. CD32 KO K562 cell line and generation of CD64 transgenic CD32 KO K562. **(B)** Flow cytometry plots showing CD16, CD32, and CD64 expression on the three cell lines. **(C)** Representative plots of human CAlleR 8 T cell proliferation after 96 h of coculture with K562 expressing CD32 or CD64 (K562:T cell ratio of 1:10) in the presence of Bet v1 (0.5 μg/ml) and different concentrations of mAb 10.

**Figure S3.**
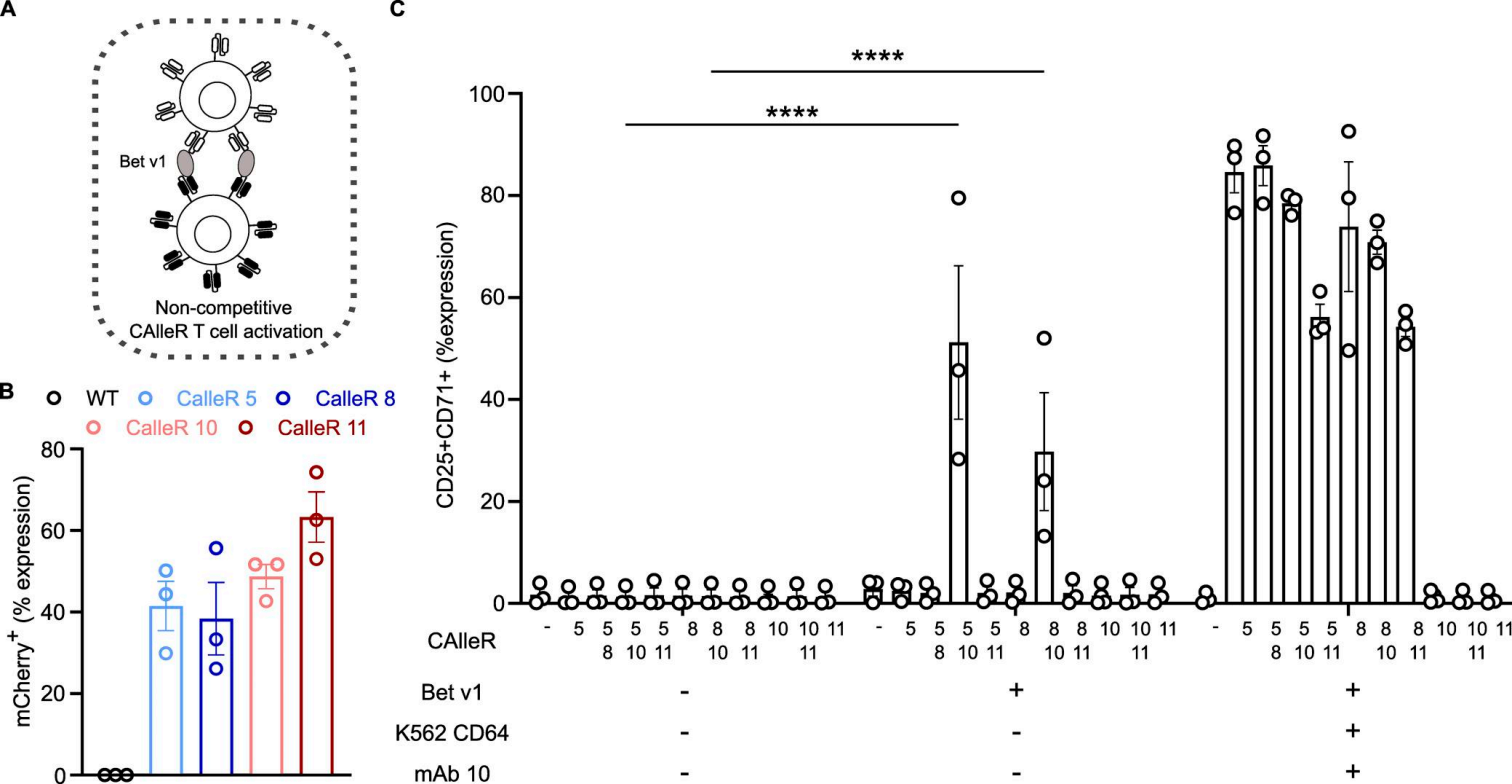
**CAlleR T cell activation by soluble Bet v1 through noncompetitive scFvs. (A)** Schematic representation of two different CAlleR T cells interacting through noncompetitive binding of the scFv to Bet v1. **(B)** Transduction efficiency of anti-Bet v1 CAlleR T cells assessed by mCherry reporter expression by flow cytometry. **(C)** Cumulative data showing percentages of CD25^+^CD71^+^ CAlleR T cells (gated on mCherry^+^) after 48-h coculture with other CAlleR T cells (1:1 ratio) in presence or absence of K562 CD64 (1:5 ratio), Bet v1 (0.5 μg/ml) and mAb 10 (1 μg/ml). Data represent three biological independent experiments and are shown as the mean ± SEM in B and C. Exact P values were determined by two-way ANOVA with Tukey’s test in C. ****P < 0.0001.

Since CAR signaling for soluble ligands relies on ligand-mediated dimerization and eukaryotic cells do not constitutively express allergens (Chang et al., 2018), we hypothesized that anti-Bet v1 antibodies can stabilize soluble antigens and contribute to CAlleR activation. Indeed, soluble Bet v1 alone was insufficient to activate CAlleR T cells (Fig. 4 A). Yet, in the presence of noncompetitive mAbs we observed a modest upregulation of activation markers (Fig. 4 A) in line with the competition binding assays (Fig. 1, G–I). To further assess the antibody-dependent activation, we examined the effect of low-, medium-, and high-affinity FcγR (CD16, CD32, and CD64, respectively) by generating specific K562 cell lines (Fig. S2, A and B). The activation of CAlleR T cells was FcγR-dependent and required the stabilization of the Bet v1 by noncompetitive birch-specific antibodies (Fig. 4 A). Importantly, we observed an antibody dose-dependent activation of the CAlleR 8 in the presence of mAb 10 (Fig. 4, B and C; and Fig. S2 C). Furthermore, two noncompetitive scFvs are sufficient to stabilize and crosslink the allergen to induce CAlleR T cell activation in the presence of soluble Bet v1 (Fig. S3, A–C). The capacity of noncompetitive Bet v1–specific antibodies to induce OX40 and 4-1BB upregulation in CAlleR Tregs was further confirmed (Fig. 4 D) and correlated with their trogocytosis capacities (Fig. 4 E). The suppressive capacity of CAlleR 8 Tregs was FcγR and antibody concentration–dependent and better than polyclonal Tregs in all conditions (Fig. 4, F and G). Interestingly, the suppression was less efficient against CAlleR 8 T cells stimulated with mAb 10 bound to CD64 compared with CD32, suggesting more resistance to Treg when effector T cells (Teff) are strongly activated (Fig. S2 C).

### CAlleR murine Teff proliferated *in vivo* in an antibody-dependent manner

**Figure 5.**
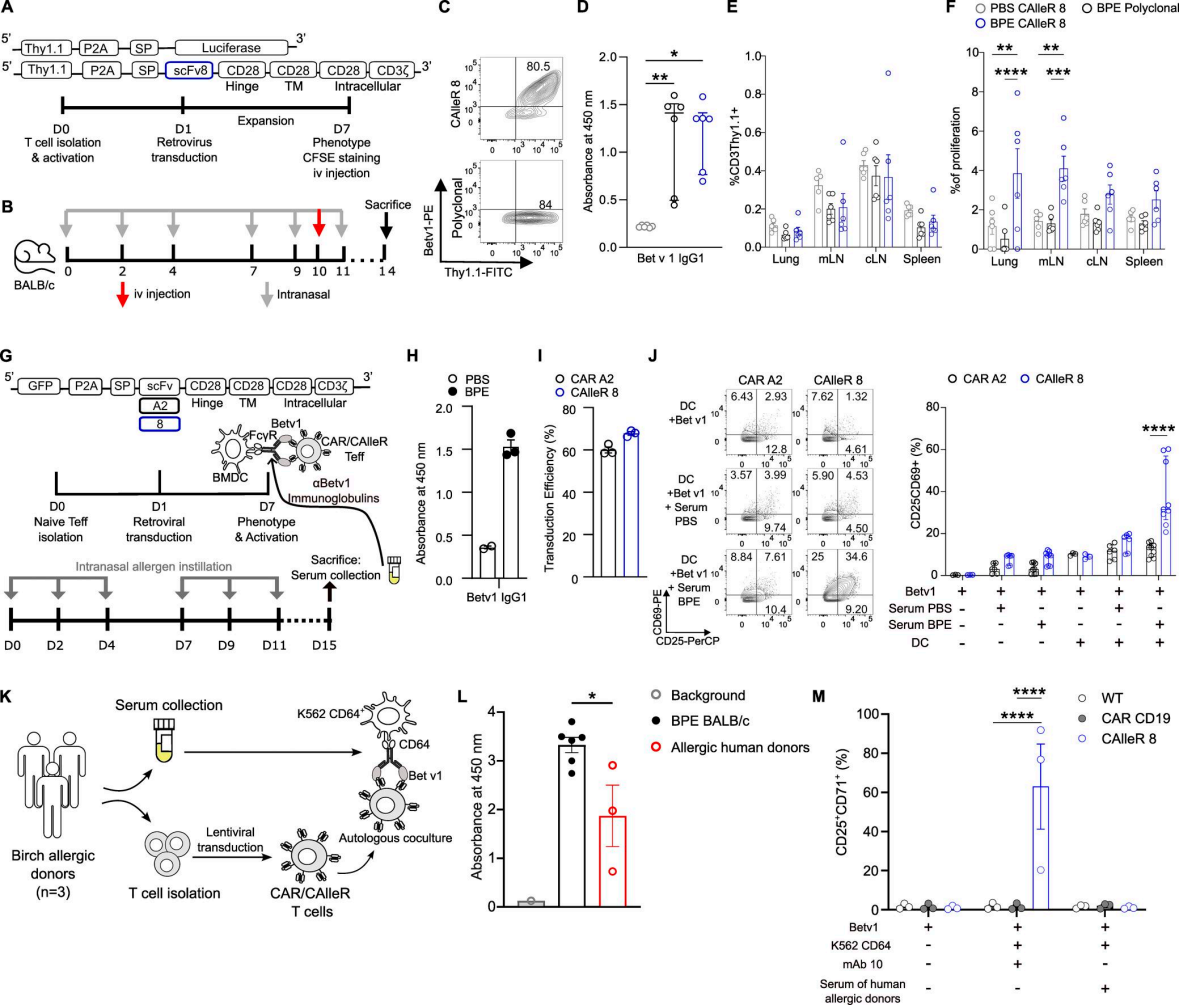
**CAlleR 8 murine T cell activation and proliferation in birch-sensitized mice. (A)** Experimental design of murine CAlleR 8 and polyclonal Teff generation. **(B)** Experimental design of the allergic airway inflammation mouse model with adoptive transfer of transduced Teff at day 10. **(C)** Transduction efficiency of Tconv after 7 days of expansion. **(D)** Bet v1 IgG1 detection in murine sera by ELISA (*n* = 6). **(E)** Cumulative data of the percentage of transduced (Thy1.1^+^) Tconv detected among the CD3^+^ cells in the different collected organs (lung, mLN, cLN, and spleen) (*n* = 5–6). **(F)** Cumulative data of the percentage of Thy1.1^+^CD4^+^ cells that proliferated (gated on CFSE^−^) in the different organs in mice exposed to PBS or BPE (*n* = 6). **(G)** Experimental design of CAR A2 and CAlleR 8 Teff production and coculture with BMDCs in the presence of Bet v1 and serum from mice exposed to either PBS or BPE. **(H)** Bet v1–specific IgG1 detection in the serum of mice exposed to PBS (*n* = 2) or BPE (*n* = 3) by ELISA. **(I)** Cumulative data of transduction efficiency (Thy1.1^+^) of CAR A2 and CAlleR 8 Teff after 7 days of expansion (*n* = 3). **(J)** Representative flow cytometry plots and cumulative data showing the expression of CD25 and CD69 in CAR A2 and CAlleR 8 Teff after 24-h coculture with Bet v1 (0.5 μg/ml), mouse serum (10%), and/or BMDCs (BMDC:Teff ratio of 1:5) (for each condition and for each serum, three biologically independent samples of Teff were tested). **(K)** Schematic representation of the experimental design. **(L)** Anti-Bet v1 IgG detection in the serum (1:500 dilution) of birch pollen–sensitized mice (*n* = 6) and allergic donors (*n* = 3). **(M)** Cumulative data showing the percentage of untransduced, CD19 CAR, and CAlleR 8 T cells expressing CD25 and CD71 after 48-h coculture with Bet v1 (0.5 μg/ml), K562 expressing CD64 (1:5 ratio), and 20% of autologous serum from allergic donors or mAb 10 (1 μg/ml) (*n* = 3 allergic donors). Data in E, F, H, I, L, and M are represented by the mean ± SEM and by median ± interquartile range in D and J. P values in D were calculated with the Kruskal–Wallis test with Dunn’s multiple comparison test, in E, F, and M with two-way ANOVA and Tukey’s multiple comparison test, in J with the Mann–Whitney U test, and in L with an unpaired *t* test. *P < 0.05; **P < 0.01; ***P < 0.001; ****P < 0.0001. cLN, cervical lymph node; Tconv, conventional T cell.

The *in vivo* functionality of the CAlleR was first validated by engineering murine Teff and monitoring CFSE dilution. CAlleR 8 Teff were infused in birch pollen extract (BPE)–sensitized mice (Fig. 5, A–D). Their presence and proliferation were compared in the lung, mediastinal lymph node (mLN), cervical lymph node, and the spleen of PBS and BPE-sensitized mice. CAlleR 8 and polyclonal Teff were detected in all organs (Fig. 5 E). Yet, CAlleR 8 Teff proliferated significantly more than their polyclonal counterpart in a birch-dependent manner, predominantly in the lungs and draining mLNs (Fig. 5 F). To confirm CAlleR 8 Teff proliferation in response to birch-specific immunoglobulins in a FcγR-dependent manner, we purified bone marrow–derived dendritic cells (BMDCs) expressing high levels of FcγR (Guilliams et al., 2014). We then investigated whether BPE-exposed mice produced specific immunoglobulins capable of mediating CAlleR 8 T cell activation via the FcγR on BMDCs (Fig. 5 G). After the sensitization protocol, high levels of anti-Bet v1 IgG1 were detected in allergic mouse serum (Fig. 5 H), which induced CAlleR 8 T cell activation when BMDCs and the allergen were present (Fig. 5, I and J). These findings demonstrate that CAlleR 8 Teff proliferate in allergen-exposed organs in a birch-specific antibody–dependent manner. A similar experimental design was applied using peripheral blood mononuclear cells (PBMCs) and serum samples from birch-allergic donors (Fig. 5 K). However, the significantly lower levels of anti-Bet v1 IgG in donor serum compared with sensitized mice (Fig. 5 L) were likely insufficient to trigger CAlleR 8 T cell activation in autologous coculture with decomplemented serum and CD64-expressing K562 cells (Fig. 5 M).

### CAlleR 8 murine Tregs suppress allergic airway inflammation in mice

**Figure 6.**
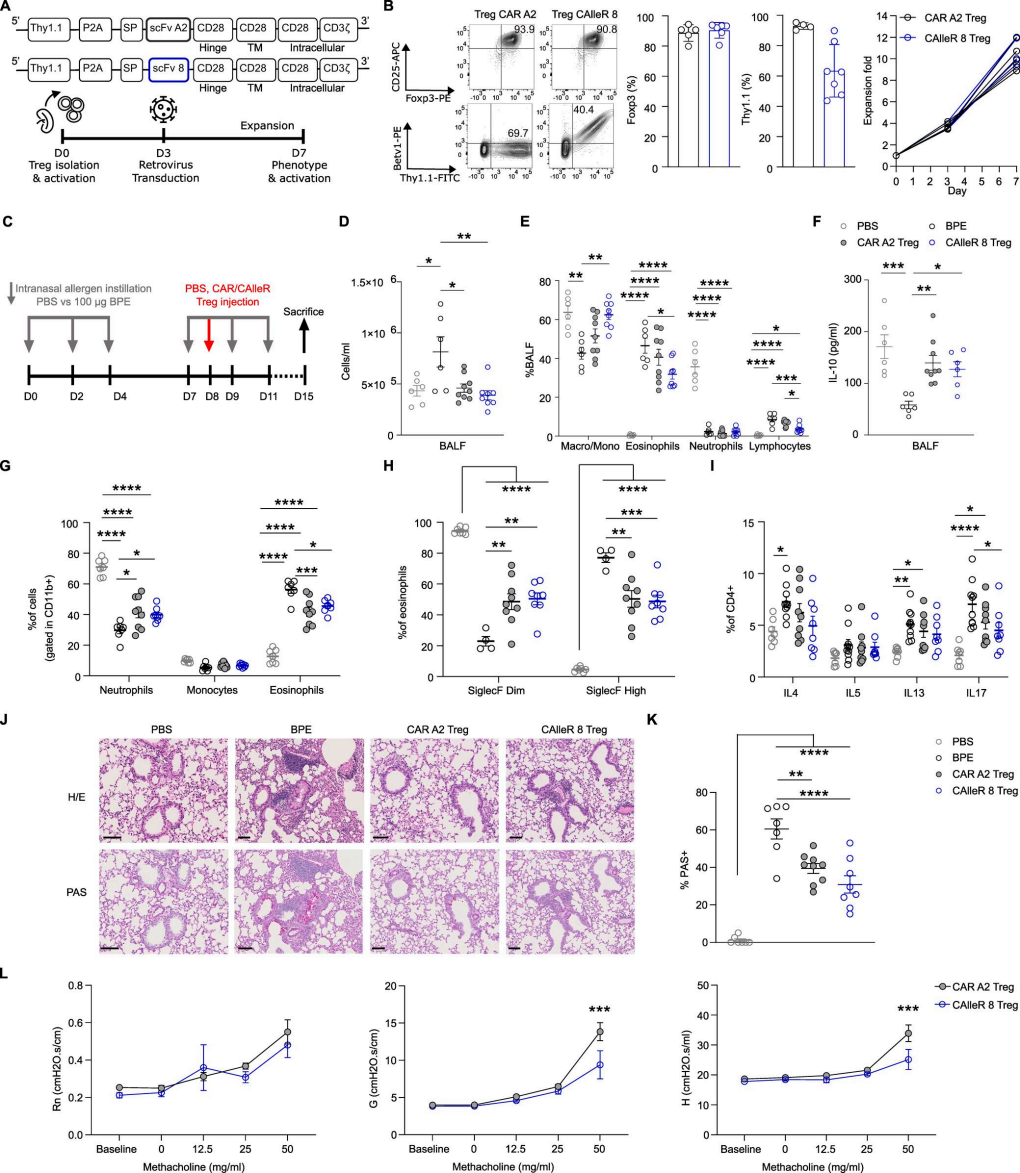
**CAlleR 8 murine Tregs for the treatment of allergic airway inflammation. (A)** CAR A2 and CAlleR 8 murine constructs. Experimental design for murine CAlleR Treg production. **(B)** Representative flow cytometry plots and cumulative data of CAlleR Treg phenotype after 7 days of expansion with CD25 and Foxp3 expression to assess purity (*n* = 4–6) and Thy1.1 and Bet v1 expression for transduction efficiency (*n* = 4–7). Cumulative data of Treg expansion after 7 days of culture. **(C)***In vivo* experimental design of BPE-induced allergic airway inflammation. **(D)** Cell counts in BALF (*n* = 6–9). **(E)** Differential counts of cells infiltrating the BALF (*n* = 6–9). **(F)** IL-10 quantification in BALF by ELISA. **(G)** Percentages of granulocytes in the lungs (*n* = 7–9). **(H)** SiglecF expression on eosinophils in the lungs (*n* = 4–9). **(I)** Th2 cytokine production in CD4^+^ T cells of the lungs (*n* = 7–10). **(J)** Representative images of H/E and PAS staining of the lungs (black bars represent 100-μm scale). **(K)** Quantification of PAS+ small and medium airways of the lungs (*n* = 7–9). **(L)** FlexiVent measurement of Newtonian resistance (Rn), tissue elastance (H), and tissue damping (G) at baseline and at different doses of inhaled methacholine (*n* = 4–5). Data are presented as the mean ± SEM from two independent experiments (except for L that shows data from one experiment). *n* indicates the number of mice. P values were determined by one-way ANOVA with Tukey’s multiple comparison test in D, E, F, G, I, and K and two-way ANOVA with Tukey’s multiple comparison test in H and Sidak’s test in L. *P < 0.05; **P < 0.01; ***P < 0.001; ****P < 0.0001. H/E, hematoxylin/eosin.

**Figure S4.**
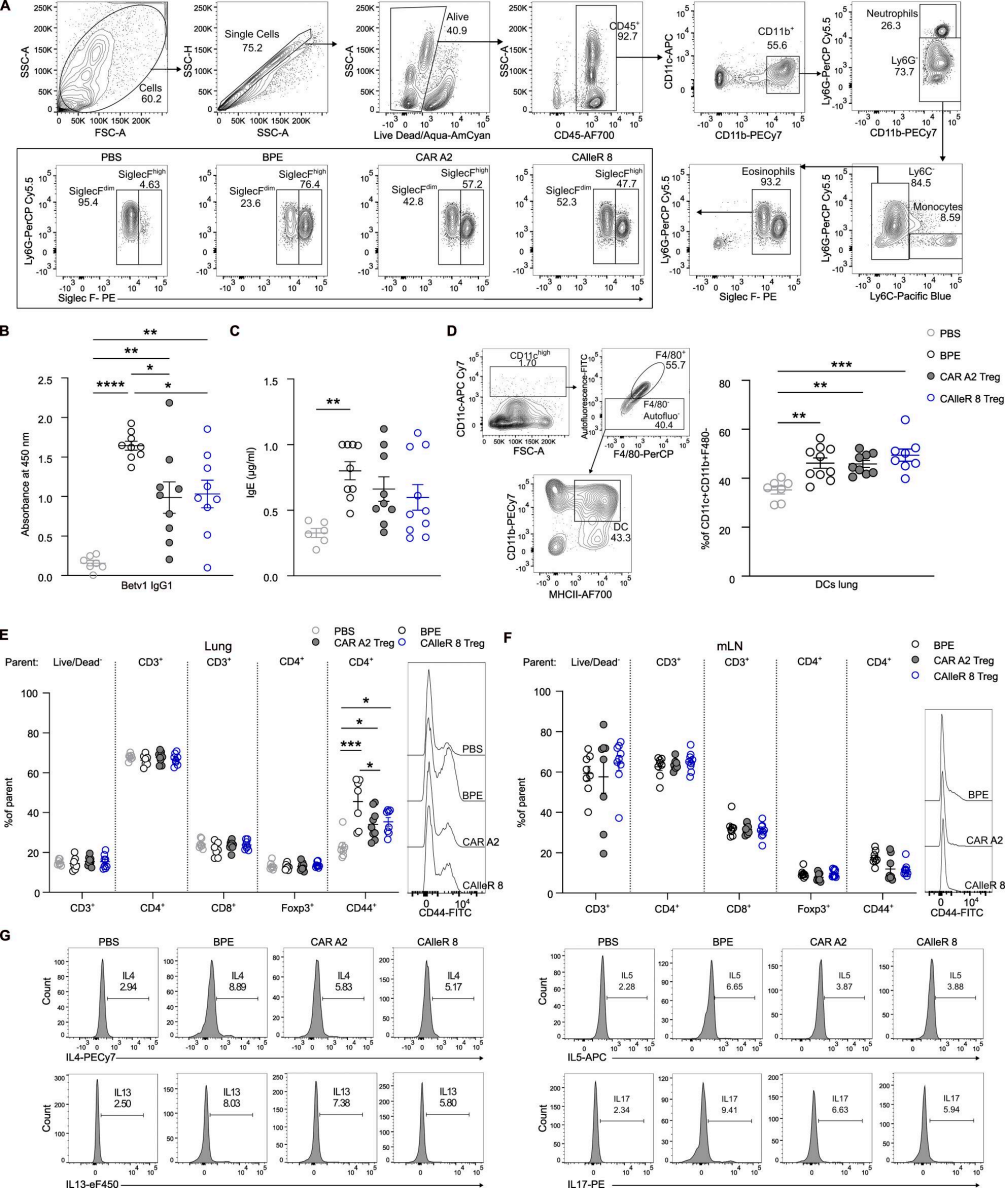
**CAlleR 8 Tregs do not regulate immunoglobulin production or affect the T cell and dendritic cell compartments in the treatment model. (A)** Representative flow cytometry plots showing the gating strategy for neutrophils, monocytes, and eosinophils in the lungs. The expression of SiglecF in lung eosinophils is also shown. **(B)** Specific anti-Bet v1 IgG1 detection in mouse serum by ELISA (*n* = 7–9 from two independent experiments). **(C)** Total serum IgE quantification by ELISA (*n* = 6–10 from two independent experiments). **(D)** Gating strategy and cumulative data on the percentage of DCs detected in the lungs (gated on CD11c^+^CD11b^+^F480^−^MHC-II^+^) (*n* = 7–10 from two independent experiments). **(E and F)** Cumulative data of the percentage of the different T populations observed in (E) the lungs and in (F) the mLN (CD3^+^ T cells, CD4^+^, CD8^+^, and Tregs) and representative plots and cumulative percentage of CD4^+^ T cells expressing the activation marker CD44 (*n* = 7–10 from two independent experiments). **(G)** Representative flow cytometry plots showing intracellular expression of Th2 cytokines in lung CD4^+^ T cells. Data are represented as the mean ± SEM, and P values were calculated with one-way ANOVA with Tukey’s multiple comparison test in B, C, and D and with two-way ANOVA with Tukey’s multiple comparison test in E and F. *P < 0.05; **P < 0.01; ***P < 0.001; ****P < 0.0001. DCs, dendritic cells.

Considering the risks associated with CAR T cell therapies, we generated CAlleR murine Tregs expressing either the CAlleR 8 or a control anti-HLA-A2 CAR (Wagner et al., 2022) with Thy1.1 as a reporter (Fig. 6 A). After 7 days of culture, phenotype and transduction efficiency were evaluated (Fig. 6 B) prior to injection to BPE-exposed mice (Fig. 6 C). Treg-treated mice had lower cell influx in the bronchoalveolar lavage fluid (BALF) than allergic mice indistinctively of their specificity (Fig. 6 D). Importantly, CAlleR 8 Tregs significantly reduced the eosinophil percentage in the BALF (Fig. 6 E). Additionally, Treg infusion correlated with increased IL-10 levels in the BALF (Fig. 6 F). In the lungs of Treg-treated mice, lower levels of eosinophils (CD45^+^CD11c^−^CD11b^+^Ly6G^−^Ly6C^−^SiglecF^+^) were detected (Fig. 6 G and Fig. S4 A). Notably, the expression of SiglecF, a marker for eosinophilic activation, was also decreased on these cells (Fig. 6 H and Fig. S4 A). These findings coincided with reduced birch-specific IgG and total IgE levels in the serum of these mice (Fig. S4, B and C). However, dendritic cell (CD11c^+^CD11b^+^F4/80^−^MHC-II^+^) infiltration in the lungs was not reduced (Fig. S4 D). Although no overall differences were observed in the proportions of T cell populations in the lungs and mLNs (Fig. S4, E and F), Treg-treated mice exhibited reduced CD4^+^ T cell activation, as indicated by reduced CD44 expression (Fig. S4 E), along with diminished production of IL-4, IL-13, and IL-17 (Fig. 6 I and Fig. S4 G). Lung mucus production was quantified using periodic acid–Schiff (PAS) staining. Mice treated with CAlleR 8 Tregs showed fewer PAS^+^ airways than the other groups (Fig. 6, J and K). This finding was supported by improved lung function with CAlleR 8 Treg–treated mice showing reduced airway hyperreactivity, as measured by lung stiffness (H) and tissue resistance (G) after methacholine challenge (Fig. 6 L). Overall, these data demonstrate the protective nature of Treg in a preclinical mouse model of allergic airway inflammation that can be further enhanced with CAlleRs.

### CAlleR 8 Tregs prevent allergic airway inflammation

**Figure 7.**
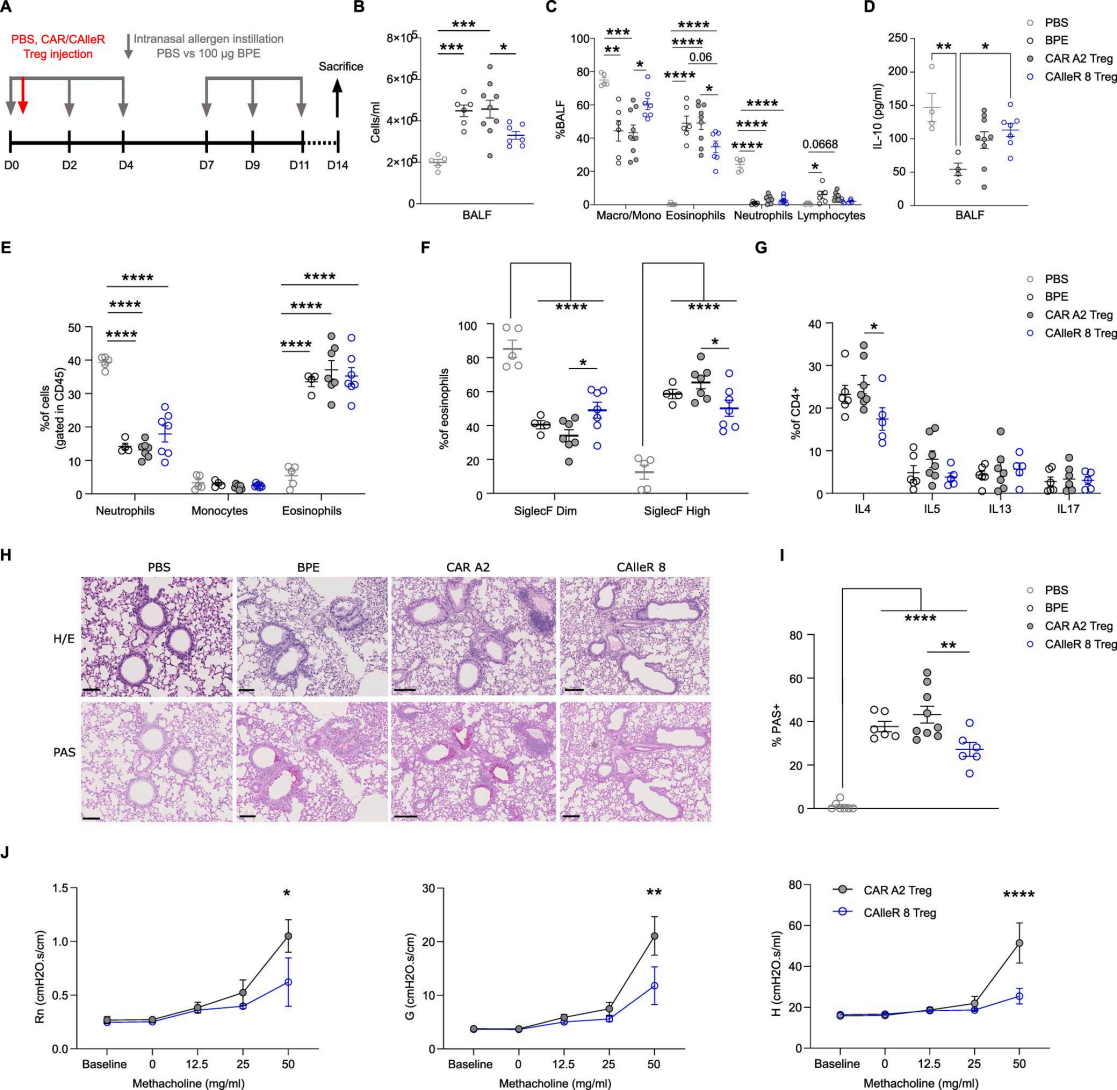
**CAlleR 8 murine Tregs for the prevention of allergic airway inflammation. (A)** *In vivo* experimental design of BPE-induced allergic airway inflammation. **(B)** Cell counts in BALF (*n* = 5–9). **(C)** Differential counts of cells infiltrating the BALF (*n* = 5–9). **(D)** IL-10 quantification in BALF by ELISA. **(E)** Percentages of granulocytes in the lungs (*n* = 4–7). **(F)** SiglecF expression on eosinophils in the lungs (*n* = 4–7). **(G)** Th2 cytokine production in CD4^+^ T cells of the mLN (*n* = 5–7). **(H)** Representative images of H/E and PAS staining of the lungs (black bars represent 100-μm scale). **(I)** Quantification of PAS^+^ small and medium airways of the lungs (*n* = 6–9). **(J)** FlexiVent measurement of Newtonian resistance (Rn), tissue elastance (H), and tissue damping (G) at baseline and at different doses of inhaled methacholine (*n* = 5). Data are presented as the mean ± SEM from two independent experiments (except for J that shows data from one experiment). P values were determined by one-way ANOVA with Tukey’s multiple comparison test in B, C, D, E, G, and I and with two-way ANOVA with Tukey’s multiple comparison test in F and Sidak’s test in J. *P < 0.05; **P < 0.01; ***P < 0.001; ****P < 0.0001. H/E, hematoxylin/eosin.

**Figure S5.**
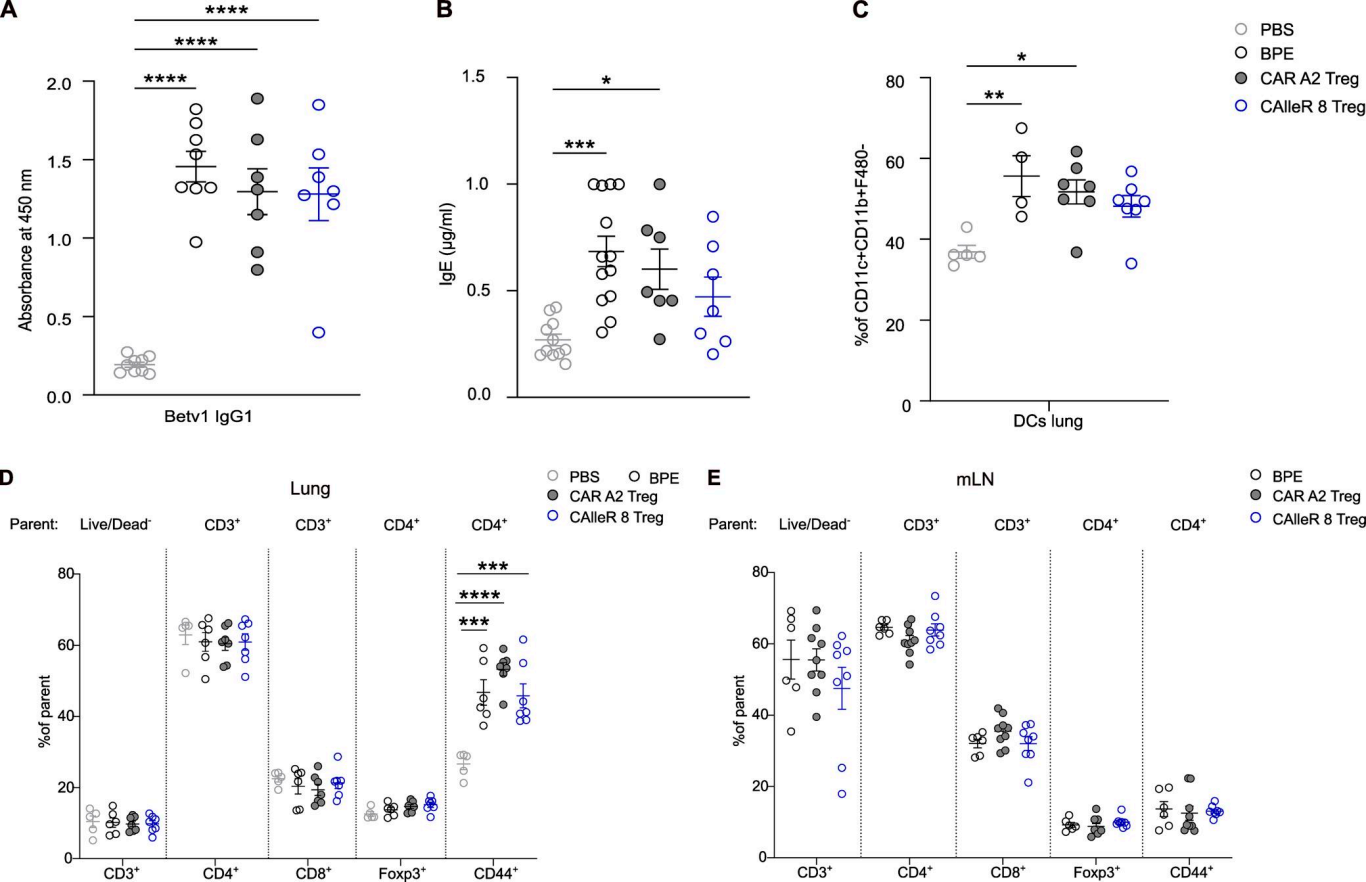
**CAlleR 8 Tregs do not prevent immunoglobulin production or the activation of T cells and DCs. (A)** Specific anti-Bet v1 IgG1 detection in mouse serum by ELISA (*n* = 4–7 from two independent experiments). **(B)** Total serum IgE quantification by ELISA (*n* = 7–13 from two independent experiments). **(C)** Cumulative data on the percentage of DCs detected in the lungs (gated on CD11c^+^CD11b^+^F480^−^MHC-II^+^) (*n* = 4–7 from two independent experiments). **(D and E)** Cumulative data of the percentage of the different T populations observed in (D) the lungs and in (E) the mLN (CD3^+^ T cells, CD4^+^, CD8^+^, and Tregs) (*n* = 5–9 from two independent experiments). Data are represented as the mean ± SEM, and P values were calculated with one-way ANOVA with Tukey’s multiple comparison test in A–C and with two-way ANOVA with Tukey’s multiple comparison test in D and E. *P < 0.05; **P < 0.01; ***P < 0.001; ****P < 0.0001. DCs, dendritic cells.

We next evaluated whether Tregs can prevent allergic disease development. To this end, engineered Tregs were adoptively transferred to mice prior to their first exposure to BPE (Fig. 7 A). In this context, only the CAlleR 8 Tregs reduced the cellular influx in the BALF, with a lower proportion of eosinophils and lymphocytes (Fig. 7, B and C). Increased IL-10 was also detected in the BALF of CAlleR 8 Treg–treated mice (Fig. 7 D). In the lung, no significant differences were observed in the proportion of neutrophils (CD45^+^CD11c^−^CD11b^+^Ly6G^+^), monocytes (CD45^+^CD11c^−^CD11b^+^Ly6G^−^Ly6C^+^), and eosinophils (CD45^+^CD11c^−^CD11b^+^Ly6G^−^Ly6C^−^SiglecF^+^) (Fig. 7 E). Yet, SiglecF high eosinophils were significantly less prevalent in mice treated with CAlleR 8 (Fig. 7 F). Although in this model the anti-Bet v1 IgG1 production remained unaffected by Treg transfer (Fig. S5 A), CAlleR 8 Tregs reduced total IgE levels (Fig. S5 B). CAlleR 8 Tregs did not significantly affect the dendritic cell (CD11c^+^CD11b^+^F4/80^−^MHC-II^+^) infiltration in the lungs (Fig. S5 C). Similarly, Treg prophylactic treatment had no effect in the T cell compartment, neither on the proportions of the different populations nor on their activation status in the lungs and the mLNs (Fig. S5, D and E). However, significantly less IL-4 production by CD4^+^ T cells was observed (Fig. 7 G). Importantly, mice treated with CAlleR 8 Tregs exhibited reduced mucus production (Fig. 7, H and I), a finding associated with significantly improved lung function with reduced airway hyperreactivity, as measured by reduced airway (Rn) and tissue (G) resistance, as well as lung stiffness (H) after methacholine challenge (Fig. 7 J). These data demonstrate that CAlleR 8 Tregs can prevent the inflammatory response of birch pollen–induced allergic asthma.

### CAlleR 8 Tregs migrate to BPE-exposed lungs and mLNs to preferentially interact with CD11c^+^ APCs

**Figure 8.**
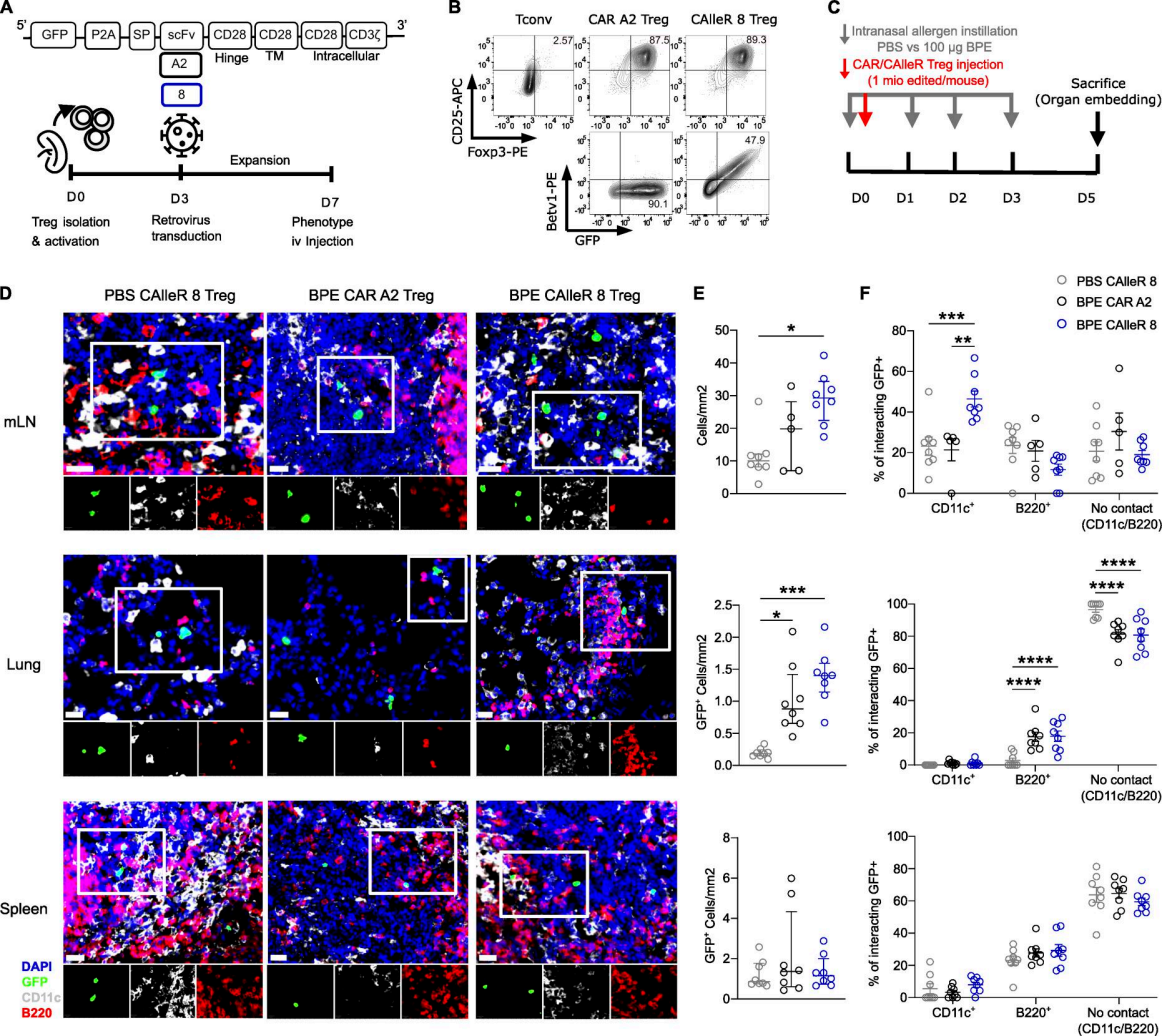
**CAlleR 8 murine Treg tissue–specific homing. (A)** Experimental design of murine CAlleR 8 GFP^+^ and CAR A2 GFP^+^ Treg production. **(B)** Representative plots of Treg phenotype and transduction efficiency after 7 days of expansion. **(C)** Experimental design of the allergic airway inflammation mouse model and adoptive Treg transfer. **(D)** Representative images of CAlleR 8 and CAR A2 Tregs in the mLNs, lung, and spleen of mice exposed to PBS or BPE. White bars represent the scale of 20 μm, and small images show fluorescence channels of the magnified region outlined in white squares. **(E)** Cumulative data of detection of GFP^+^ cells/mm2 in each organ (*n* = 8 in the lung and spleen and *n* = 5–8 in mLN from four mice with image duplicates). **(F)** Cumulative data of the percentage of GFP^+^ cells interacting with CD11c^+^, B220^+^ APCs or with none of them (no CD11c^+^/B220^+^ contact) for each organ (*n* = 8 in the lung and spleen and *n* = 5–8 in the mLN from four mice with image duplicates). In E, data are represented by the median ± interquartile range and in F by the mean ± SEM. P values were calculated with Kruskal–Wallis and Dunn’s multiple comparison test in E and with two-way ANOVA with Tukey’s test in F. *P < 0.05; **P < 0.01; ***P < 0.001; ****P < 0.0001.

To further investigate the mechanism of CAlleR Treg *in vivo*, we engineered the cells with a GFP reporter (Fig. 8 A). After *in vitro* expansion, transduction and phenotype of the engineered Tregs were confirmed (Fig. 8 B) and cells were adoptively transferred to mice, which were then exposed to BPE for four consecutive days (Fig. 8 C). In mice exposed to BPE, the number of CAlleR 8 Tregs was significantly higher in the lungs and draining lymph nodes (mLN) compared with PBS control mice (Fig. 8, D and E). Importantly, CAlleR 8 Tregs made significantly more contacts with CD11c^+^ APCs present in the mLN than unspecific A2-CAR Tregs (Fig. 8 F). This difference was not observed in the lung and spleen of the mice (Fig. 8 F). Thus, the CAlleR enabled specific migration and interactions of the Tregs with CD11c^+^ APCs present in the mLN.

## Discussion

Herein, we provide proof-of-concept and preclinical evidence that CAlleR Treg redirected against Bet v1 can downmodulate birch pollen–induced allergic airway inflammation. While polyclonal, allergen-unspecific Tregs demonstrated partial protective effects under inflammatory conditions, they were insufficient to prevent disease onset in the absence of inflammation. Importantly, we identified a new mechanism underlying the activation of CAR T cells targeting soluble antigens: engagement through high-affinity FcγRs recruiting antibodies, which are specific for a noncompetitive epitope that preserves the binding site of the CAR. This mechanism opens new avenues not only for rewiring synthetic receptors against any soluble antigens including autoantigens for therapeutic intervention but also for delineating a more global pathway for antigen cross-presentation in allergies. Several attempts have been made to develop CAR T therapies targeting soluble factors, such as mesothelin ligand (Lanitis et al., 2012), carcinoembryonic antigen (Nolan et al., 1999), factor VIII (Fu et al., 2020), Lewis-Y carbohydrate antigen (Westwood et al., 2009), BCMA (Carpenter et al., 2013), CD30 (Hombach et al., 1998), or insulin (Tenspolde et al., 2019) with limited success. In fact, soluble antigens in their monomeric form, as for nominal Bet v1 antigen, consistently failed to induce CAR T cell activation (Chang et al., 2018). In contrast, dimeric soluble ligands, such as TGF-β, can cluster CARs together and mediate T cell responsiveness, an actin-dependent dynamic process relying on mechanical forces between the targeted antigen and the CAR (Chang et al., 2018; Qiu et al., 2024). In this study, we discovered that CAlleR T cells can respond to soluble Bet v1 in a FcγR-dependent manner when specific antibodies not interfering with the CAR (noncompetitive) are present. As high titer of allergen-specific IgGs was detectable in birch-sensitized mice, this mechanism of allergen presentation could indeed contribute in the recruitment and activation of CAlleR Tregs. One could also speculate on the existence of a similar cross-presentation pathway with allergen-specific B cell receptor. Thus, the relevance of this mechanism warrants further investigation, which should also include a prospective and longitudinal monitoring of sensitized patients during on- and off-pollen period season.

Our data indicated a preferential migration of CAlleR Tregs to the mLNs and the lungs, reinforcing initial observations that redirecting the specificity of Tregs is sufficient to retain them in various tissues, such as the gut (Porret et al., 2025) or an HLA-A2 islet graft (Muller et al., 2021a). Yet, in inflammatory conditions, i.e., during the second round of allergen sensitization, we also observed a functional activity of the nonspecific CAR-edited Tregs. While the HLA-A2 CAR remains inactive in HLA-A2–negative control mice, thus effectively ruling out off-target activity (Wagner et al., 2022), its enhanced recruitment in the lung may be associated with the pulmonary passage following cell infusion (Fischer et al., 2009), which, in combination with pro-inflammatory chemokines, promotes homing and persistence of the cells in the lungs. Imprinting of Tregs by lung dendritic cells could favor CCR4 upregulation and thereby a more efficient trafficking to the lungs (Mikhak et al., 2013). Afshar et al. demonstrated that CCR7 on Treg is required to suppress allergic airway inflammation during the sensitization phase, whereas CCR4 is essential for suppressing inflammation during the effector phase (Afshar et al., 2013). Future studies should better characterize the interplay between the CAlleR and the chemokine receptors in the trafficking dynamics between the mLN and the lung.

Our data build on previous evidence suggesting that the transfer of Tregs can modulate allergic airway inflammation *in vivo* (Kearley et al., 2005; Xu et al., 2012). The main advantage of Tregs over other cell therapy modalities, such as CAR T cells redirected against IL-5 and secreting IL-4/13 muteins (Jin et al., 2024), is their safety profile, as they lack proliferative capacities and produce considerably less pro-inflammatory cytokines (Esensten et al., 2018). Our findings show that CAlleR Tregs reduced levels of IL-4 in CD4^+^ T cells, which could explain the decrease of eosinophils in the BALF as IL-4 mediates eosinophils’ transendothelial migration (Moser et al., 1992). Beyond their immunoregulatory functions, Tregs also contribute directly to tissue repair and homeostasis through amphiregulin-dependent mechanisms, an additional remarkable advantage over more conventional T cell therapies (Arpaia et al., 2015). Yet, bona fide self-antigen–specific Tregs can lose Foxp3 expression during an inflammatory autoimmune response (Bailey-Bucktrout et al., 2013) or upon repetitive stimulation (Hoffmann et al., 2009). Thus, future studies should evaluate the persistence and stability of the CAlleR Tregs over time and define the optimal modalities for implementing such approach, e.g., outside the pollen season or as an adjunct to a desensitization protocol to reduce local inflammation.

The present study has several limitations. First, we focused on birch pollen–related allergic airway inflammation. However, patients with severe allergic asthma often suffer from multiple sensitizations against PR-10–unrelated proteins, such as profilins or polcalcins (Cacheiro-Llaguno et al., 2024; Ruiz-Hornillos et al., 2020). Therefore, future studies should evaluate the potential cross-reactive and bystander suppression of birch pollen–specific CAlleR Tregs and their capacity to suppress PR-10–related and unrelated memory T cells, respectively. Second, the *in vivo* effects of the CAlleR Tregs remain modest compared with other cell therapy products, such as IL-5–targeting CAR T cells (Jin et al., 2024). While the security profile of Treg-based therapy is certainly higher, optimized CAlleR designs combining several complementary allergen–specific scFvs may be necessary to improve their responsiveness to soluble allergens. Importantly, future studies should evaluate the response of CAlleR Tregs from allergic patients during and outside the pollen seasons. Third, redirecting Treg specificity toward perennial allergens such as house dust mite may be also relevant considering the challenges associated with allergen avoidance. Accordingly, future studies should investigate the suppressive capacity of CAlleR Tregs in polysensitized mouse models. In parallel, it will be critical to assess the phenotypic stability and functional activities of CAlleR Tregs under repeated allergen stimulation as we have not investigated their persistence beyond 5 days after injection. Also, we did not assess the suppressive capacity of Tregs on allergen-specific B cells, which may also contribute to allergen mobilization through their BCRs. Finally, future studies should evaluate whether FcγR-dependent allergen cross-presentation is also relevant in other cell types, including B cells, as well as basophils and mast cells.

In conclusion, we demonstrate that CAlleR Tregs can effectively prevent and downmodulate birch pollen–induced allergic airway inflammation. Our data provide compelling evidence for the role of noncompetitive “allosteric” antibodies in enhancing the action and homing of Tregs, contributing to the restoration of immune tolerance to soluble allergens. Future work should evaluate whether such approach could also be suitable to restore tolerance against food allergies.

## Materials and methods

### Human blood products

Deidentified human peripheral blood from healthy donors was ordered at the Swiss Transfusion Center as buffy coats. For B cell sorting, PBMCs were isolated from a birch-allergic female donor enrolled in the Immuno-IgE study. The study was approved by the Institutional Review Board of the Lausanne University Hospital (2020-02798; CER-VD, Switzerland). Written and informed consent was obtained prior to sample collection. Ficoll-Paque (Cat. #17144003; Cytiva) density gradient centrifugation was used to isolate PBMCs.

### B cell sorting, immortalization, and cloning

B cells were isolated from PBMCs with EasySep Human B Cell Isolation Kit according to the manufacturer’s instructions (Cat. #17954; StemCell Technologies) and labeled with LIVE/DEAD Fixable Aqua Dead Cell Stain Kit (Cat. #L34957; Invitrogen), mouse anti-human CD19 APC (Cat. #555415; BD Biosciences; clone SJ25C1), mouse anti-human IgM-PECy7 (Cat. #314532; BioLegend; clone MHM-88), mouse anti-human IgG AF488 (Cat. #410706; BioLegend; clone M1310G05), mouse anti-human IgE PE (Cat. #325506; BioLegend; clone MHE-18), mouse anti-human CD27 (Cat. #646851; BD Biosciences; clone HB7). Viable CD19^+^CD27^+^IgM^−^IgG^+^IgE^−^ B cells were sorted with BD FACSAria II cell sorter (BD Biosciences). B cell immortalization was performed as previously described (Traggiai et al., 2004). Briefly, B cells were incubated for 3 h at 37°C in complete DMEM (Cat. #A4192102; Thermo Fisher Scientific) supplemented with 10% FCS, 1% penicillin–streptomycin, 1% MEM nonessential amino acids (Cat. #11140050; Thermo Fisher Scientific), 1% L-glutamine, 1% Na-pyruvate, 30 μg/ml transferrin (HOLO) (Cat. #7542-100; Biovision), 1% kanamycin, 55 μM 2-mercaptoethanol (Cat. #21985-023; Gibco), 2 μg/ml CL264 TLR-7 agonist (Cat. #tlrl-c264e-5; InvivoGen), and 40% Epstein-Barr virus obtained from human gammaherpesvirus 4 cell’s supernatant (Cat. #VR-1492; ATCC). B cells were plated at three cells per well with 26,000 autologous irradiated PBMCs in a 384-well plate. Screening for each well was performed with an indirect Bet v1 ELISA detecting anti-Bet v1 antibodies in the supernatant. Among 5780 clones, we could identify 21 binders, which were further selected for RT-PCR as previously described (Fenwick et al., 2021). Among those 21 clones, 4 IgG and 3 IgE paired heavy and light variable chain productive sequences were obtained with Sanger sequencing (Fasteris). PCR amplification with custom primers (Microsynth AG) was performed to extract the variable chain sequences of the antibodies and for subcloning into AbVec2.0-IGHG1, AbVec1.1-IGKC, or AbVec1.1-IGLC2-XhoI vectors (#80795, #80796, and #99575; Addgene plasmids, respectively). Resulting plasmids were used for transient transfection of ExpiCHO cells with 1.5 μg/ml of each plasmid. After 7 days of cell culture, supernatants were collected and antibodies were purified using Sartobind Lab protein A columns according to the manufacturer’s instructions (Cat. #93PRAP06HB-12--A; Sartorius AG).

### Recombinant Bet v1 production

The Bet v1 DNA sequence was codon-optimized for *Escherichia coli*, synthesized by GenScript, and cloned into a bacterial expression vector, pET29b (#110168; Addgene, RRID:Addgene_110168), with a 10xHis-tag at the C terminus and an AviTag. Recombinant Bet v1 (rBet v1) was expressed in *E. coli* BL21(DE3) cells by growing cells at 37°C until an OD of 0.6, followed by induction with 0.5 mM isopropyl β-D-1-thiogalactopyranoside overnight at 18°C. The bacterial pellet was harvested for 15 min at 5,000*g*, and was lysed using sonication in buffer A (20 mM HEPES 7.5, 700 mM NaCl, 10% glycerol). Clarification of the cell’s lysate was performed by centrifugation for 30 min at 30,000*g*. The protein was purified by nickel affinity, with a HisTrap HP 5 ml column, washed with buffer A, and then eluted on a linear gradient of buffer B (buffer A + 500 mM imidazole, pH 7.5). Eluted fractions of rBet v1 were concentrated and injected onto a Superdex 75 16/600 gel filtration column (GE Healthcare/Cytiva) in 20 mM HEPES 7.5, 250 mM NaCl. After size exclusion, PBS buffer exchange was performed by dialysis and rBet v1 was diluted at 1 mg/ml and stored at −20°C.

### ELISA

Antibody binding toward Bet v1 was assessed by indirect ELISA. NuncSorp plates were coated with 2 μg/ml of rBet v1 diluted in coating buffer (15 mM Na_2_CO_3_, 34.87 mM NaHCO_3_) overnight at 4°C, washed with PBS/Tween 0.05%, and blocked with PBS-BSA 1% for 2 h at room temperature (RT). Serial dilutions of the seven novel mAbs were performed starting at 1 μg/ml and incubated for 2 h. Serum samples from mice were diluted at 1:100 prior to incubation. Plates were then washed, and 1 μg/ml of biotin mouse anti-human IgG antibody (Cat. #555785; BD Pharmingen; clone G18-14, RRID:AB_396120), biotin mouse anti-human IgE antibody (Cat. #325504; BioLegend; clone MHE18, RRID:AB_830849), or 2 μg/ml goat anti-mouse IgG1 cross-adsorbed secondary antibody, Biotin-XX (Cat. #A10519; Life Technologies, RRID:AB_10374477) was used, respectively, for detection and incubated for 1 h at RT. Streptavidin–horseradish peroxidase conjugate (Cat. #554066; BD Pharmingen; 1:1,000 dilution) was added and incubated for 1 h at RT. Tetramethylbenzidine substrate (Cat. #555214; BD Biosciences) was added for 20 min, and the reaction was stopped with 2N sulfuric acid. Absorbance was measured on a spectrophotometer at 450 nm (630 nm reference). Binding of mAbs to PR-10 allergens (rMal d1 [Bühlmann Laboratories AG], rCor a1 [Cat. #RE-CA101-1; Inbio], and rGly m4 [Cat. #RP-GM4-1; Inbio] and to other non-PR-10 allergens [kindly provided by Dr. Régine Audran [Lausanne University Hospital, Lausanne, Switzerland] and Dr. Craig Fenwick [Lausanne University Hospital, Lausanne, Switzerland] [rag weed, grass, Derp2, bee venom]) was assessed using the same protocol, coating PR-10 allergens at 2 μg/ml and non-PR-10 allergens at 10 μg/ml. For total quantification of murine IgE, purified goat anti-mouse IgE antibody (Cat. #1110-1; SouthernBiotech, RRID: AB_2794601) was coated at 2 μg/ml in PBS and incubated overnight at 4°C. After washing and blocking, 1:50 dilution of serum samples and twofold serial dilutions of purified mouse IgE for standard curve starting at 2 μg/ml (Cat. #553481; BD Pharmingen, RRID: AB_10050441) were added to the wells. After 2 h of incubation, wells were washed and detection goat anti-mouse IgE-AP antibody was added at 2 μg/ml (Cat. #1110-04; SouthernBiotech, RRID: AB_2794603) and incubated for 2 h. Finally, wells were washed and 4-nitrophenyl phosphate disodium salt hexahydrate (Cat. #N2765-100TAB; Sigma-Aldrich) was diluted in diethanolamine buffer (1M, pH 9.8, Cat. #31590-250G; Sigma-Aldrich), added to the wells, and incubated for at least 5 min prior to absorbance measurement at 450 nm. IL-10 ELISA was performed according to the manufacturer’s instructions (ELISA MAX Standard Set Mouse IL-10, Cat. #431411; BioLegend).

### Hierarchical clustering of mAbs

Heavy and light chain amino acid sequences were aligned using ANARCI (Dunbar and Deane, 2016) with the IMGT numbering scheme, and concatenated per antibody. Pairwise sequence similarity was calculated as 1 minus the normalized Hamming distance between concatenated sequences. The resulting distance matrix was hierarchically clustered and visualized as a dendrogram using the ComplexHeatmap package (Gu et al., 2016).

### Affinity and blocking capacity of anti-Bet v1 antibodies

Affinity toward Bet v1 for each antibody was assessed with Gator Prime Core BLI System by using a protein A–coated probe (Cat. #160001; Gator). After binding of the antibodies to the biosensors, tips were dipped into 500 or 300 nM of the Bet v1 protein. The strength of association and dissociation of the Bet v1 to the antibodies was measured.

Blocking capacity of the anti-Bet v1 antibodies was assessed as previously described (Atanasio et al., 2022) by coating NuncSorp plates with 2 μg/ml of anti-human IgE mAb (clone Le27; Cat. #0908-1-010; NBS-C BioScience) in coating buffer and incubated overnight at 4°C. Washing with PBS/0.05% Tween and blocking with PBS/1%BSA for 2 h at RT were performed before adding the serum of birch-allergic patients diluted in dilution buffer so that the concentration added to the wells was 4 ng/ml of anti-Bet v1 IgE. Biotinylated rBet v1 at 1 nM was premixed for 2 h at RT with threefold serial dilutions of the four anti-Bet v1 antibodies starting at 1 μM and then added to the IgE-coated plate for 2 h at RT. Plates were subsequently washed, and streptavidin–horseradish peroxidase conjugate (Cat. #554066; BD Pharmingen; 1:1,000 dilution) was added and incubated for 1 h at RT. Tetramethylbenzidine substrate (Cat. #555214; BD Biosciences) was added for 20 min, and the reaction was stopped with 2N sulfuric acid. Absorbance was measured on a spectrophotometer at 450 nm (630 nm reference). Calculation of the percentage of blocking was performed as follows:

$$\begin{array}{c} \%\;\text{of Blocking}=100\times \\ \frac{\text{Absorbance without αBet v1 Ab}-\text{Absorbance with αBet v1 Ab}}{\text{Absorbance without αBet v1 Ab}} \end{array}$$

### Competition assay with Luminex bead–based assay

Covalent coupling of rBet v1 to Luminex beads was performed according to the manufacturer’s instructions with BioPlex Amine Coupling Kit (Cat. #171406001; Bio-Rad). 30-fold molar excess of capture mAbs was combined with rBet v1–coupled beads for 30 min. Biotinylated competitor mAbs were then added to each well and incubated for further 20 min. Biotinylated mAbs bound to rBet v1 were stained with streptavidin-PE (Cat. #554061; BD Pharmingen; 1:1,000 dilution) and analyzed on 200 BioPlex instruments.

### Cryo-EM data collection

Grids were screened for particle presence and ice quality on a TFS Glacios microscope (200 kV), and the best grids were transferred to a TFS Titan Krios G4. Cryo-EM data were collected using a TFS Titan Krios G4 transmission electron microscope, equipped with a Cold-FEG on a Falcon IV detector in electron counting mode. Falcon IV gain references were collected just before data collection. Data were collected using TFS EPU version 2.12.1 utilizing the aberration-free image shift protocol, recording four micrographs per ice hole. Movies were recorded at a magnification of 120,000×, corresponding to the 0.658 Å pixel size at the specimen level, with defocus values ranging from −1 to −2.4 µm. Exposures were obtained with 60 e^−^/Å^2^ total dose. In total, 4,530 micrographs in EER format were collected.

### Cryo-EM data processing and structure fitting

Data processing was performed with cryoSPARC (version 4.4) including motion correction and contrast transfer function (CTF) determination. Particle picking and extraction (extraction box size 256 pixels) were carried out using cryoSPARC version 4.478. Next, several rounds of reference-free 2D classification were performed to remove artifacts and selected particles were used for ab initio reconstruction and heterorefinement. After heterorefinement, 18,497 particles contributed to an initial 3D reconstruction of 4.8 Å resolution (Fourier shell coefficient 0.143) with a C1 symmetry. A model of a Bet v1 (PDB ID 4A81) and AlphaFold2 (ColabFold implementation) models of the Fab08 and Fab10 were fitted into the cryo-EM maps with UCSF ChimeraX (version 1.5). These docked models were extended and rebuilt without lateral chain manually using Coot (version 0.9.8.8) and Phenix (version 1.21) (Emsley et al., 2010; Liebschner et al., 2019). Figures were prepared using ChimeraX (USCF) (Goddard et al., 2018).

### Cell line generation

NFAT-Luciferase Jurkat cells were purchased from BPS Bioscience Inc. (#60621) and cultured in RPMI 1640 (Gibco) supplemented with 10% FBS, 1% nonessential amino acids, 1% sodium pyruvate, 1% penicillin–streptomycin, and geneticin (1 mg/ml). Lentiviruses encoding the different anti-Bet v1 CAlleRs or the anti-CD19 CAR with mCherry reporter were used to transduce cells that were further sorted to purify mCherry^+^ cells. For K562 Bet v1 generation, the Bet v1 protein linked to the PDGF transmembrane domain and to GFP was cloned in the lentiviral expression vector pCDH-EF1-FHC plasmid (#64874; Addgene, RRID: Addgene_64874) (Yousefzadeh et al., 2014) under the EF1a promoter. Lentiviral particles were used to transduce tumor K562 cell line (RRID: CVCL_0063) that was further sorted to purify GFP^+^ cells. K562 cells were genetically modified to knock out CD32 (FcγRII) using CRISPR-Cas9. Briefly, 80 μM CRISPR RNA (crRNA) sequences targeting FCGR2A gene (5′-TGG​AGC​ACG​TTG​ATC​CAC​GG-3′ and 5′-AAA​GCA​CAG​TCA​GAT​GCA​CA-3′) (Synthego) and 80 μM trans-activating crRNA (Lot# 748524; IDT) were complexed with 45 μM high-fidelity recombinant Cas9 protein (produced by the Protein Production and Structure Core Facility of the EPFL—Swiss Federal Technology Institute of Lausanne, Switzerland) and mixed with 1 × 10^6^ K562 cells resuspended in 20 μl Lonza P3 Primary Cell Solution prior to electroporation in Lonza 4D-Nucleofector X Unit with the FF-120 program. After electroporation, cells were immediately transferred to prewarmed RPMI-1640 medium supplemented with 10% FBS and incubated at 37°C, 5% CO_2_. CD32-deficient cells were further sorted. To generate K562 CD64 cells, CD32-deficient cells were transduced with a lentivirus encoding human CD64 and further sorted.

All cell lines were checked for *Mycoplasma* contamination with negative results.

### Animals

Female BALB/cByJ mice aged 6 wk were purchased from Jackson Laboratory (RRID: IMSR_JAX:001026) and maintained at the animal facility of the University of Lausanne. All animal protocols were approved by the Cantonal Commission for Animal Experiments from the Canton of Vaud (Switzerland) (license number: VD3893).

### Primary cell preparation, isolation, and sorting

Human T cells were enriched using EasySep Human T Cell Enrichment Kit following the manufacturer’s instructions (Cat. #17952; StemCell Technologies). For human Tregs, CD25^+^ PBMCs were enriched using CD25 MicroBeads II (Cat. #130-092-983; Miltenyi Biotec) and stained with CD4-FITC (BD), CD25(CD25-4E3)-APC, and CD127-PE for further sorting on a BD FACSAria II cell sorter (BD Biosciences) as CD4^+^CD25^+^CD127^low^. Human primary cells were cultured for 2 days in X-Vivo 15 medium (Cat. #02-053Q; Lonza) supplemented with 5% human AB serum (Cat. #P30-2901; Pan-Biotech), 1% penicillin–streptomycin (Cat. #4-01F00-H; BioConcept), 55 μM 2-mercaptoethanol (Cat. #21985-023; Gibco), and 10 mM N-acetyl-L-cysteine (Cat. #A9165-25G; Sigma-Aldrich). After viral transduction, cells were cultured in RPMI medium (Cat. #61870-010; Gibco) supplemented with 10% FBS (Cat. #F7524; Sigma-Aldrich), 1% nonessential amino acids (Cat. #11140-050; Gibco), 10 mM HEPES (Cat. #15630-056; Gibco), 1 mM sodium pyruvate (Cat. #11360-039; Gibco), and 1% penicillin–streptomycin. Medium was supplemented with recombinant human IL-2 (Cat. #130-097-746; Miltenyi Biotec) at 30 IU/ml for Teff and 300 IU/ml for Tregs. Both T cells and Tregs were activated from day 0 with anti-CD3/CD28 Dynabeads (Cat. #11131D; Gibco) at 1:1 ratio.

Murine T cells were isolated from spleens and lymph nodes of female BALB/c mice. T cells were enriched by negative selection using EasySep Mouse T Cell Isolation Kit (Cat. #19851; StemCell Technologies). For Treg isolation, CD4^+^ T cells were isolated using EasySep CD4^+^ T Cell Isolation Kit according to the manufacturer’s instructions (Cat. #19852A; StemCell Technologies). CD4^+^ T cells were stained with CD25(PC61)-PE (Invitrogen) and CD4(RM4-5)-APC (BD), and the CD4^+^CD25^high^ fraction was purified using MoFlo Astrios (Beckman Coulter) or Aria II (BD) cell sorters. Murine T cells were cultured in supplemented RPMI cell culture medium with 55 μM 2-mercaptoethanol (Cat. #21985-023; Gibco). T cells were activated with mouse anti-CD3/CD28 Dynabeads (Cat. #11452D; Gibco) at 1:1 bead-to-cell ratio for conventional T cells and at 3:1 ratio for Tregs. The medium was supplemented with 50 IU/ml human IL-2 (Cat. #130-097-746; Miltenyi Biotec), 5 ng/ml human IL-7 (Cat. #130-095-362; Miltenyi Biotec), and human 5 ng/ml IL-15 (Cat. #130-095-764; Miltenyi Biotec) for conventional T cells. For Tregs, medium was supplemented with 2000 IU/ml recombinant human IL-2 (Cat. #130-097-746; Miltenyi Biotec) for Treg. On day 7 of culture, beads were removed and cells rested overnight before further experiments.

### Plasmids

Second-generation CARs/CAlleRs with the CD28 hinge, CD28 transmembrane, CD28 intracellular domains, and the CD3ζ signaling domain were cloned in the pCDH-EF1-FHC lentiviral vector plasmid (no. 64874; Addgene) as previously described (Muller et al., 2021a). The CARs were designed using the scFv derived from the four novel anti-Bet v1 antibodies and the anti-CD19 scFv derived from clone FMC63 as previously described (Muller et al., 2021b).

The murine stem cell virus–based splice-gag vector (pMSGV) was used to introduce the scFv of either the CAlleR 8 or an anti-HLA A2 CAR (clone SN607D8) (Muller et al., 2021a; Wagner et al., 2022) fused to the hinge, transmembrane, and intracellular domains of mouse CD28, and the signaling domain of mouse CD3ζ. The retroviral vector was kindly provided by Dr. Melita Irving (Lausanne University Hospital and University of Lausanne, Lausanne, Switzerland).

### Virus production

To produce lentiviruses, 3 × 10^6^ HEK293T cells were seeded in 9 ml of supplemented DMEM and transfected 24 h later with 4 μg of vector plasmid, 4 μg of pCMV-dR8.9 packaging plasmid, and 2 μg of pMD2.G2 packaging vector diluted in PEI. The supernatant was collected 48 and 72 h after transfection, filtered through a 45-μm filter, ultracentrifuged at 50,000*g* for 2 h, and stored at −80°C.

Retroviruses were produced using Platinum-E (Plat-E) Retroviral Packaging Cell Line (Cat. #RV-101; Cell Biolabs). Briefly, Plat-E cells were grown in DMEM supplemented with 10 μg/ml blasticidin (Cat. #ant-bl-1; InvivoGen) and 1 μg/ml puromycin (Cat. #P8833; Sigma-Aldrich) before transfection with 10 μg of CAR and 10 μg of pCL-Eco (#12371; Addgene plasmid, RRID:Addgene_12371) in Turbofect reagent (Cat. #R0531; Thermo Fisher Scientific). Two days after transfection, Plat-E supernatant was collected, and viruses were concentrated 400 times after ultracentrifugation at 50,000*g* for 1 h 30 min at 4°C.

### Cell transduction

For human cells, transduction was performed 24 h after activation by adding a MOI of 1 of pretitrated lentivirus to the culture. After 20 h of incubation, the virus was washed, and cells were resuspended in fresh supplemented medium with cytokines. For murine cells, transduction was performed 24 h after activation for conventional T cells and 72 h after activation for Tregs. Cells were incubated with 10% concentrated retrovirus in a nontreated culture plate (Greiner Bio-One) precoated overnight with 20 µg/ml RetroNectin (Cat. #T100A; Takara Bio). The cell–virus mixture was spinoculated at 2,000*g* for 1.5 h at 32°C, followed by overnight incubation at 37°C, 5% CO_2_. After 18 h of transduction, the virus was removed by washing, and cells were resuspended in fresh medium.

### Luciferase assay

To screen CAlleR functionality, NFAT cells were cocultured with K562 WT or K562 expressing Bet v1 at 2:1 ratio for 24 h at 37°C, 5% CO_2_. After incubation, the luciferase assay was performed as previously described^23^. Briefly, cells were lysed for 20 min under shaking conditions with 50 μl harvesting buffer (50 mM 2-morpholinoethanesulfonic acid sodium [NaMES] [Cat. #1061970100; Sigma-Aldrich], 50 mM Tris-HCl, 1 mM dithiothreitol [Cat. #R0861; Thermo Fisher Scientific], 0.4% Triton X-100 [Cat. #9002-93-1; Thermo Fisher Scientific]). After incubation, 50 μl luciferase buffer (125 mM NaMES, 125 mM Tris-HCl, 25 mM [CH_3_COO]_2_Mg · 4H_2_O [Cat. #M0631; Sigma-Aldrich], 2.5 mM adenosine triphosphate [ATP, Cat. #R0441; Thermo Fisher Scientific]) was added and incubated for 1 min under shaking conditions prior to adding 50 μl of luciferin buffer (1 mM D-luciferin [Cat. #88292; Thermo Fisher Scientific] in 4.8 mM KH_2_PO_4_ [Cat. #1.04873; Merck] and incubating for 5 min protected from light. Luminescence was measured as relative light units on the Synergy H1 Hybrid reader [BioTek]).

### *In vitro* human T cell and Treg activation, proliferation, and suppression assays

For activation and proliferation assays involving human primary T cells and Tregs, 0.1 × 10^6^ primary cells were cocultured with 120 G irradiated K562 Bet v1 at different ratios, or when not specified at 1:5 ratio. For activation assay testing different FcγRs, 120 G irradiated K562 at 1:5 ratio was used in the presence of the Bet v1 protein (1 μg/ml), anti-Bet v1 mAbs (0.5 μg/ml), and/or decomplemented serum from birch-allergic donors. After 2 days of coculture, cells were stained for activation markers and analyzed by flow cytometry. Anti-CD3 was used as a positive control (1 μg/ml, clone OKT3, Cat. #566685; BD Biosciences). For activation of CAlleR T cells cocultured with other CAlleR T cells, 1:1 ratio was used in the presence of Bet v1 (0.5 μg/ml).

For suppression assays, Tregs were debeaded, washed, rested for 8 h, and plated in 1:2 serial dilutions up to 1:64. Beads were also removed from Teff, and cells were then washed and stained with 1 μM CFSE (Vybrant CFDA SE Cell Tracer Kit, Cat. #V12883; Invitrogen) in PBS for 5 min. Then, 0.1 × 10^6^ Teff were cocultured with Tregs at different ratios and 0.01 × 10^6^ irradiated K562 cells. Suppression assays using different FcγR K562 cell lines, Tregs, and Teff were plated as previously mentioned using 0.5 μg/ml of Bet v1 and different concentrations of mAb 10. Cells were incubated for 96 h before analyzing CFSE dilutions. FlowJo software was used to calculate the division index, and the percentage of suppression for each condition was calculated as follows:

$$\text{Suppression}\;\left(\%\right)=\left(1-\frac{\text{division}\;\text{index}\;\left(\text{condition}\right)}{\text{division}\;\text{index}\;\left(\text{positive}\;\text{control}\right)}\right)\times \;100$$

Each replicate of activation and suppression assays corresponded to an independent donor.

### *In vitro* murine T cells and Treg activation

*In vitro* activation of CAlleR murine cells was assessed by coculturing 0.1 × 10^6^ T cells or Tregs with 0.02 × 10^6^ BMDCs generated as previously described (Alcaraz-Serna et al., 2021) in the presence of Bet v1 (0.5 μg/ml) and 10% heat-inactivated murine serum. Cells were incubated for 24 h, and upregulation of activation markers was assessed by flow cytometry.

### Mouse model of birch pollen–induced allergic airway inflammation

BPE (European white Betula pendula, Cat. #XP527D3A25; Stallergenes Greer) was reconstituted in sterile PBS at 3.33 mg/ml. On days 0, 2, 4, 7, 9, and 11, mice were anesthetized by inhalation in a chamber with 4% isoflurane for 1 min and intranasally instilled with 30 μl of diluted BPE. On day 0 (prophylactic model) or on day 8 (treatment model), mice were randomly assigned to receive adoptive transfer of CAR or CAlleR murine Tregs or vehicle (PBS). On days 14 or 15, mice were euthanized with 225 mg/kg intraperitoneal injection of pentobarbital (Esconarkon, Streuli Pharma AG).

### Organ collection and processing

After sacrifice, blood was collected from abdominal aorta for serum isolation. Lungs were extracted, cut, and digested for 30 min at 37°C with 0.2 mg/ml of collagenase D (Cat. #11088858001; Sigma-Aldrich) resuspended in HBSS (Gibco), 10 mM HEPES, and 5% FBS. mLNs were collected and digested the same way as lungs. Organs were then smashed on a 70-μm cell strainer with syringe plungers and stained for flow cytometry analysis. Spleens were directly smashed on 70-μm cell strainers, and erythrocytes were lysed with ACK lysis buffer (155 mM NH_4_Cl, 10 mM KHCO_3_, 0.1 mM EDTA in distilled water, pH 7.4) prior to staining for flow cytometry.

For BALF collection, tracheas were cannulated with an 18 G Venflon (Cat. #393226; BD) and flushed three times with 500 μl of ice-cold PBS supplemented with 0.2% BSA (Cat. #A8806; Sigma-Aldrich). Total BALF cell counts were determined with Coulter Counter Multisizer 4e (Cat. #B23005; Beckman Coulter). Cytospins were obtained by centrifuging 80,000 cells at 800 rpm for 5 min. Resulting cytospin slides were stained with RAL Diff-Quik solution (Cat. #720555-0000; CellaVision). Percentages of monocytes/macrophages, eosinophils, neutrophils, and lymphocytes were assessed by counting 200 cells per cytospin.

### Flow cytometry

For human samples, cells were washed once with PBS supplemented with 0.5% FBS, 0.4% EDTA. After discarding supernatant, cells were incubated for 30 min at 4°C in the same wash buffer with diluted antibodies: anti-human CD3 (UCHT-1), PECy7 (1:400 dilution), 563423; BD Biosciences, RRID:AB_2738196; anti-human CD4 (RPA-T4), AF700 (1:400 dilution), 557922; BD Biosciences; anti-human CD8 (RPA-T8), APC-Cy7 (1:400 dilution), 557760; BD Biosciences; anti-human CD8 (SK1), PE (1:250 dilution), 345773; BD Biosciences; anti-human CD25 (M-A251), FITC (1:400 dilution), 555431; BD Biosciences; anti-human CD25 (CD25-4E3), APC (1:250 dilution), 17-0257-42; Thermo Fisher Scientific; anti-human CD64 (REA978), APC (1:200 dilution), 130-116-197; Miltenyi Biotech; anti-human CD71 (M-A712), FITC (1:400 dilution), 555536; BD Pharmingen; anti-human CD127 (hIL-7R-M21), PE (1:200 dilution), 557938; BD Biosciences; anti-human CD32 (FLI8.26), APC (1:200 dilution), 559769; BD Biosciences; anti-human CD16 (3G8), PE (1:200 dilution), 555407; BD Biosciences; biotinylated rBet v1 (1 μg/ml); streptavidin, PE (1:1,000 dilution), 554061; BD Biosciences; DAPI (Live/Dead), Invitrogen (1:2,500 dilution), D1306; Phantom Dye (Live/Dead), Pacific Blue (1:2,500 dilution), PD00004; Proteintech. After 30-min incubation, samples were washed again and either resuspended in washing buffer prior to flow cytometry acquisition, or permeabilized and fixed using eBioscience Foxp3/Transcription Factor Staining Buffer according to the manufacturer’s instructions (Cat. #00-5523-00; Invitrogen). Intracellular staining was performed with anti-human Foxp3 (PCH101), eFluor660 (1:250 dilution), 50-4776-42; Thermo Fisher Scientific; Helios (22F6), PE (1:250 dilution), 137216; BioLegend.

For murine samples, cells were incubated with an anti-mouse CD16/32 antibody (TruStain FcX PLUS, clone S17011E; BioLegend (1:250 dilution), Cat. #156604, RRID:AB_2783138) for 15 min at 4°C. After blocking, cells were washed and extracellular staining was performed for 30 min at 4°C with the following antibodies: anti-mouse CD3 (145-2C11), PE-Cy7 (1:250 dilution) and APC-Cy7 (1:100 dilution), 552774; BD Biosciences (RRID:AB_394460) and 561042 (RRID:AB_2034003), respectively; anti-mouse CD4 (RM4-5), PerCP-Cy5.5 (1:300 dilution) and APC (1:250 dilution), 561090 and 553051; BD Biosciences, respectively; anti-mouse CD8a (53-6.7), FITC (1:100 dilution) and Pacific Blue (1:400 dilution), 553030 and 558106; BD Biosciences, respectively; anti-mouse CD25 (PC61.5), PE (1:200 dilution), 12-0251-83; Invitrogen; anti-mouse CD25 (PC61), PerCP-Cy5.5 (1:250 dilution), 551071; BD Biosciences; anti-mouse CD44 (IM7), FITC (1:100 dilution), 553133; BD Biosciences; anti-mouse CD45 (30-F11), PE-Cy7 (1:250), 552848; BD Biosciences; anti-mouse CD45.2 (104), AF700 (1:100 dilution), 109821; BioLegend; anti-mouse CD69 (H1.2F3), PE (1:400 dilution), 553237; BD Biosciences; anti-mouse CD86 (GL1), PE (1:250 dilution), 553692; BD Biosciences; anti-mouse CD90.1 (Thy1.1; OX-7), PerCP-Cy5.5 (1:250 dilution), 557266; BD Biosciences; anti-mouse CD90.1 (Thy1.1; REA838), FITC (1:500 dilution), 130-112-872; Miltenyi Biotech; anti-mouse CD11c (HL3), APC (1:100 dilution), 550261; BD Biosciences; anti-mouse CD11c (N418), APC-Cy7 (1:600 dilution), 117323; BioLegend; anti-mouse CD11b (M1/70), PECy7 (1:600 dilution), 561098; BD Biosciences; anti-mouse MHC-II (M5/114.15.2), AF700 (1:1,000 dilution), 107621; BioLegend; anti-mouse F480 (BM8), PerCP (1:100 dilution), 123126; BioLegend; anti-mouse Ly6C (HK1.4), Pacific Blue (1:1,000 dilution), 128013; BioLegend; anti-mouse SiglecF (E50-2440), PE (1:100 dilution), 562068; BD Biosciences; anti-mouse Ly6G (1A8), PerCP Cy5.5 (1:100 dilution), 560602; BD Biosciences; biotinylated rBet v1 (1 μg/ml); streptavidin, APC (1:500 dilution) and PE (1:1,000 dilution), 554067 and 554061; BD Biosciences, respectively; Aqua (Live/Dead), AmCyan (1:1,000 dilution), L34957; Invitrogen; DAPI (Live/Dead), Invitrogen (1:2,500 dilution), D1306. After incubation, samples were washed again and either fixed with BD FACS Lysing Solution following the manufacturer’s instructions (Cat. #349202; BD Biosciences) or permeabilized and fixed using eBioscience Foxp3/Transcription Factor Staining Buffer set (Cat. #00-5523-00; Invitrogen) prior to intracellular staining. Intracellular staining was performed with anti-mouse Foxp3 (FJK-16 s), FITC (1:250 dilution) and PE (1:250 dilution), 11-5773-82 and 12-5773-82; Invitrogen, respectively; anti-mouse IL-4 (BVD6-24G2), PECy7 (1:100 dilution), 25-7042-41; Invitrogen; anti-mouse IL-5 (TRFK5), APC (1:100 dilution), 562048; BD Biosciences; anti-mouse IL-13 (eBio13A), ef450 (1:100 dilution), 48-7133-80; Invitrogen; anti-mouse IL-17 (eBio17B7), PE (1:100 dilution), 12-7177-81; Invitrogen.

Single-cell suspensions were resuspended in supplemented PBS prior to acquisition by an LSRFortessa cell analyzer (BD Biosciences). Flow cytometry data were analyzed using FlowJo software version 10.9.0.

### Lung function

Mice were anesthetized with intraperitoneal injection of 50 mg/kg of pentobarbital and intramuscular injection of 100 mg/kg ketamine. Mice were tracheotomized, and a 18 G metallic cannula was inserted in the trachea and secured with sutures. Mice were connected to the FlexiVent FX (SCIREQ Scientific Respiratory Equipment Inc.) and mechanically ventilated at 150 breaths/min, a tidal volume of 10 ml/kg, and a PEEP set at 3 cm H_2_O. Forced oscillation perturbation was performed to test the lung to a standardized signal of oscillatory frequencies above and below the normal ventilation frequency. Changes in resistance to increasing concentrations of nebulized acetyl-b-methacholine chloride (Sigma-Aldrich) were measured from snapshot perturbation measurements taken using the forced oscillation perturbation technique.

### Immunofluorescence organ staining, imaging, and analysis

Mice were euthanized, and lungs, mLNs, and spleen were dissected and fixed in 4% paraformaldehyde (PFA, Cat. #158127; Sigma-Aldrich) overnight at 4°C under shaking conditions. Organs were then washed three times in PBS and incubated in PBS with 30% sucrose overnight at 4°C prior to embedding in Tissue-Tek (Cat. #4583; Sakura) and snap-frozen in dry ice. Organ sections were cut with 10 μm thickness and assembled in microscopy slides for further staining. Slides were fixed in 4% PFA for 5 min, washed three times with room-temperature PBS, and incubated at RT in blocking buffer (PBS supplemented with 5% donkey serum, 0.5% BSA, 0.1% Triton X-100, 0.01% sodium azide) for 30 min. Primary antibodies (rabbit anti-GFP [Cat. #ab290; Abcam, RRID:AB_303395; 1:2,000 dilution], rat anti-mouse B220 [Cat. #14-0452-81; Thermo Fisher Scientific, RRID:AB_467253; 1:600 dilution], armenian hamster anti-mouse CD11c [Cat. #550283; BD Biosciences; 1:200 dilution]) were diluted in blocking buffer and incubated overnight at 4°C. Slides were then washed with PBS/0.3% Triton X-100, and secondary antibodies (IgG donkey anti-rabbit-AF488 [Cat. #A-21206; Thermo Fisher Scientific, RRID:AB_2535792; 1:300 dilution], IgG chicken anti-rat-AF647 [Cat. #A-21472; Thermo Fisher Scientific, RRID:AB_2535875; 1:300 dilution], IgG goat anti-armenian hamster-biotinylated [Cat. #127-065-160; Jackson Immuno Research, RRID:AB_2338980; 1:600 dilution]) were diluted in blocking buffer and incubated for 1 h at RT. After secondary staining, washing step was repeated three additional times and streptavidin-AF555 (Cat. #S32355; Thermo Fisher Scientific; 1:300 dilution) was diluted in blocking buffer, added to the slides, and incubated for 1 h at RT. Slides were washed again and mounted with Fluoromount-G Mounting Medium with DAPI (Cat. #00-4959-52; Thermo Fisher Scientific) prior to storage at 4°C.

Images were acquired at 20× magnification with a Hamamatsu NanoZoomer S60 slide scanner and analyzed with QuPath software version 0.5.1 (Bankhead et al., 2017). Briefly, whole-slide images were imported into the software, and after necessary adjustments to color normalization, regions of interest (ROIs) were manually annotated to analyze whole stained tissues. A machine learning–based classifier was developed in QuPath to differentiate positive and negative cells for each channel. Classifier’s performance was improved by refining the training dataset and adjusting classifier parameters. Then, automated cell detection was performed using QuPath’s built-in cell detection algorithm. The trained classifier was then applied to the detected cells, categorizing them as positive or negative based on the learned features. The number of GFP^+^ cells was calculated for each ROI, and density of cells was assessed by dividing the number of detected positive cells by the area of ROI. Cell interactions were quantified by determining the number of double-positive cells.

### Statistical analysis

For experiments involving human primary cells, each replicate corresponded to a unique healthy donor. In animal experiments, each replicate represented a single mouse, except for immunofluorescence staining, where two separate slides per organ were independently prepared, stained, and analyzed as technical replicates. Statistical analyses were performed using GraphPad Prism version 10.4.1 (GraphPad Software). The specific statistical tests used for each experiment are detailed in the corresponding figure legends, along with exact P values. Normality of the data was assessed using the Shapiro–Wilk test.

### Online supplemental material

Fig. S1 supports Fig. 1 by providing human B cell sorting strategy for antibody discovery and characterization of three novel human anti-Bet v1 IgE antibodies. Fig. S2 supports Fig. 4 by showing the generation and validation of K562 cell lines expressing different FcγRs. Fig. S3 supports Fig. 4 by demonstrating CAlleR T cell activation by soluble Bet v1 through noncompetitive scFvs. Fig. S4 supports Fig. 6 by showing additional experiments performed in the *in vivo* treatment model. Fig. S5 supports Fig. 7 by showing additional experiments from the *in vivo* preventive model.

## Data Availability

Data shown in the article and supplementary information are available upon reasonable request. The accession numbers for the sequences of all novel anti-Bet v1 antibodies reported in this study are GenBank: PZ143166–PZ143179. Cryo-EM map is available on Zenodo (https:/doi.org/10.5281/zenodo.16575186) and will be made public upon publication.
